# Structural Rigidity and Protein Thermostability in Variants of Lipase A from *Bacillus subtilis*

**DOI:** 10.1371/journal.pone.0130289

**Published:** 2015-07-06

**Authors:** Prakash Chandra Rathi, Karl-Erich Jaeger, Holger Gohlke

**Affiliations:** 1 Institute of Pharmaceutical and Medical Chemistry, Heinrich-Heine-University, Düsseldorf, Germany; 2 Institute of Molecular Enzyme Technology, Heinrich-Heine-University, Düsseldorf, Germany; 3 Institute of Bio- and Geosciences IBG-1: Biotechnology, Research Centre Jülich, Jülich, Germany; National Institute for Medical Research, Medical Research Council, London, UNITED KINGDOM

## Abstract

Understanding the origin of thermostability is of fundamental importance in protein biochemistry. Opposing views on increased or decreased structural rigidity of the folded state have been put forward in this context. They have been related to differences in the temporal resolution of experiments and computations that probe atomic mobility. Here, we find a significant (*p* = 0.004) and fair (*R* ^2^ = 0.46) correlation between the structural rigidity of a well-characterized set of 16 mutants of lipase A from *Bacillus subtilis* (*Bs*LipA) and their thermodynamic thermostability. We apply the rigidity theory-based Constraint Network Analysis (CNA) approach, analyzing directly and in a time-independent manner the statics of the *Bs*LipA mutants. We carefully validate the CNA results on macroscopic and microscopic experimental observables and probe for their sensitivity with respect to input structures. Furthermore, we introduce a robust, local stability measure for predicting thermodynamic thermostability. Our results complement work that showed for pairs of homologous proteins that raising the structural stability is the most common way to obtain a higher thermostability. Furthermore, they demonstrate that related series of mutants with only a small number of mutations can be successfully analyzed by CNA, which suggests that CNA can be applied prospectively in rational protein design aimed at higher thermodynamic thermostability.

## Introduction

Sufficiently high thermostability of proteins is important for both organisms living in high temperature environments and for biotechnological applications where enzymes are used as biocatalysts under often harsh reaction conditions [1, 2]. From a mechanistic point of view, “protein thermostability” embraces at least two different meanings [3, 4]: (1) *thermodynamic* thermostability describes the folded-unfolded equilibrium of a protein, and (2) *kinetic* thermostability refers to the length of time a protein remains active before undergoing irreversible denaturation at an elevated temperature. Several factors have been frequently attributed to elevated protein thermostability including improved hydrogen bonding [5], ion pair and salt bridge networks [6], better hydrophobic packing [7], shortened loops [8], and higher secondary structure content [9], in all favoring an increased structural rigidity of the folded state [10–13]. As an opposing view, proteins from thermophilic organisms have been reported to be as flexible as or even more flexible than homologs from mesophilic organisms [14–17].

These different views on the relation between protein thermostability and structural rigidity have been a matter of ongoing discussion [10, 18–23]. In particular, it has been argued that atomic movements, which are the primary mobility data from which information on protein statics (rigidity and flexibility) is derived, cover a wide range of timescales within a protein [15, 24, 25]. Hence, depending on the temporal resolution of the experimental technique or computational analysis used to detect such movements, (parts of) a protein can come out as rigid or flexible [26–32]. Here, we address the question of the relation between protein thermostability and structural rigidity by analyzing *directly* the static properties of a well-characterized set of 16 mutants of lipase A from *Bacillus subtilis* (*Bs*LipA). We do so by applying the rigidity theory-based Constraint Network Analysis (CNA) approach developed by us [33–35], thereby considering the *Bs*LipA variants to be in static equilibrium, hence avoiding that the results depend on the temporal resolution of the approach.

*Bs*LipA is an important member of the lipase class of enzymes and used in diverse biotechnological applications [36, 37]. Owing to its importance, *Bs*LipA has been extensively studied with respect to structure [38–41] and thermostability [42–48]. As to the latter, Reetz *et al*. applied iterative saturation mutagenesis on the most flexible amino acids as identified by crystallographic B-factors, which resulted in *Bs*LipA mutants that were more thermostable than the wild type showing an increase in *T*_50_^60^ (the temperature required to reduce the initial enzymatic activity by 50% within 60 min) of ≤ 45 K [42]. Subsequent biophysical characterization of the three most thermostable mutants revealed that the improved activity retention resulted from a reduced rate of protein unfolding and a reduced precipitation of the unfolding intermediates, i.e., due to kinetic reasons [49]. In contrast, Rao *et al*. sequentially developed several thermostable *Bs*LipA mutants using directed evolution assisted by structural information. These mutants were shown to be more thermostable than the wild type due to predominantly thermodynamic reasons [44–48, 50]; the most thermostable mutant displayed an increase in the melting temperature *T*_m_ of ~22 K.

In the CNA approach, a protein is modeled as a constraint network where bodies (representing atoms) are connected by sets of bars (constraints, representing covalent and noncovalent interactions) [51]. A rigidity analysis performed on the network [52, 53] results in a decomposition into rigid parts and flexible links in between. By analyzing a series of “perturbed” networks in which noncovalent interactions are included in a temperature-dependent manner [11, 13, 54], the loss of rigidity of a protein is simulated, which can be related to thermal unfolding [12, 13, 54]. Results of these analyses can be linked to biologically relevant characteristics of a biomolecular structure by a set of global and local indices [55]. In particular, a phase transition point *T*_p_ can be identified during the thermal unfolding simulation at which a largely rigid network becomes almost flexible; this phase transition point has been related to the thermodynamic thermostability of a protein [11–13]. For improving the robustness of the analyses, the rigidity analyses are performed on ensembles of network topologies (ENT^FNC^) [56]. That way, thermal fluctuations of a protein are considered without actually sampling conformations.

The main outcome of this work is the finding of a significant and good correlation between the structural rigidity of all *Bs*LipA variants and their thermodynamic thermostability. On the way, we carefully probed for the sensitivity of the results with respect to the input structures and developed an approach for detecting outliers based on differences in the pathways of thermal unfolding. We furthermore introduced a local stability measure for predicting thermodynamic thermostability, which complements the detection of the (global) phase transition point *T*_p_. As the *Bs*LipA variants are sequentially closely related, these results have important implications for applying CNA in a prospective manner in rational protein design aimed at higher thermodynamic thermostability. Finally, we discuss our results in terms of potentially different mechanisms underlying the increased protein thermostabilities of mutants isolated by Reetz *et al*.and Rao *et al*.

## Materials and Methods

### Data set

The wild type structure of *Bs*LipA with the highest resolution (PDB ID: 1ISP; resolution = 1.3 Å) was obtained from the Protein Data Bank (PDB; www.pdb.org) [57]. For probing the sensitivity of the CNA results on the conformation of the input structures, five additional crystal structures of wild type *Bs*LipA were analyzed (PDB IDs: 1I6W, 1R4Z, 1R50, 2QXT, 2QXU). We included in our study all mutants from Rao *et al*. for which *T*_*m*_ values were determined [44–48]. In addition, we included the three most thermostable mutants developed in the last rounds of iterative saturation mutagenesis by Reetz *et al*. [42]. Models of mutant structures for which crystal structures were not available in the PDB were generated with the SCWRL program [58], using the respective *Bs*LipA structure as a template that is closest in sequence to the mutant. SCWRL constructs mutant models by predicting backbone-dependent side chain conformations with the help of a rotamer library; coordinates of backbone atoms remain unchanged. Conformations of side chains of all residues within 8 Å of a mutated residue were re-predicted in order to allow for a local structural relaxation. For all structures, hydrogen atoms were added using REDUCE [59]; side chains of Asn, Gln, and His were flipped in this stage if necessary to optimize the hydrogen bond network. All water molecules, buffer ions, and crystal solvents were removed from the structures. Finally, all structures were minimized by 5000 steps of conjugate gradient minimization (including an initial steepest descent minimization for 100 steps) or until the root mean-square gradient of the energy was < 1.0·10^−4^ kcal mol^-1^ Å^-1^. The energy minimization was carried out with Amber11 [60] using the Cornell *et al*. force field [61] with modifications for proteins (ff99SB) [62] and the GB^OBC^ generalized Born model [63]. All variants of *Bs*LipA used in this study are summarized in Table 1.

**Table 1:** Summary of *Bs*LipA variants used in the study.

*Bs*LipA variant^[^ ^a^ ^]^	PDB ID^[^ ^b^ ^]^	Resolution^[^ ^c^ ^]^	Mutations	*T*_*m*_ (K)	*T*_i_ (K) ^[^ ^d^ ^]^	*T*_50_ (K)	^${\tilde{rc}}_{ij,neighbor}$ [^ ^e^ ^]^		Reference
							(kcal mol^-1^)	(K)	
Wild type	1ISP	1.3	−	329.15	324.95	321.15^[^ ^f^ ^]^	-0.87(-0.80)^[^ ^h^ ^]^	317.4 (315.9) ^[^ ^h^ ^]^	[39, 42, 44]
IX	1ISP*	−	K112D, M134D, Y139C, I157M	−	318.75	335.95^[^ ^g^ ^]^	-0.67	313.5	[42, 49]
X	1ISP*	−	R33Q, D34N, K35D, K112D, M134D, Y139C, I157M	−	321.65	362.15^[^ ^f^ ^]^	-0.73	314.5	[42, 49]
XI	1ISP*	−	R33G, K112D, M134D, Y139C, I157M	−	322.45	366.15^[^ ^f^ ^]^	-0.74	314.9	[42, 49]
TM	1T2N	1.8	L114P, A132D, N166Y	334.35	−	−	-0.88	317.6	[44]
1-14F5	1T2N*	-	TM + N89Y	336.15	−	−	-0.98	319.5	[44]
1-17A4	3D2A	1.73	TM + I157M	336.55	−	−	-1.00	319.9	[44]
1-8D5	1T2N*	−	TM + F17S	337.55	−	−	-0.81	316.3	[44]
2D9	3D2B	1.95	TM + F17S, N89Y, I157M	340.55	−	−	-0.98	319.7	[44]
3-18G4	3D2B*	−	2D9 + G111D	341.55	−	−	-0.92	318.5	[44]
3-11G1	3D2B*	−	2D9 + A20E	341.75	−	−	-0.98	319.6	[44]
3-3A9	3D2B*	−	2D9 + A15S	341.85	−	−	-0.87	317.5	[44]
4D3	3D2C	2.18	2D9 + A15S, A20E,G111D	344.35	−	−	-1.18	323.6	[44]
5-D	3D2C*	−	4D3 + S163P	345.35	−	−	-1.00	320.0	[45]
5-A	3D2C*	−	4D3 + M134E	346.05	−	−	-0.97	319.4	[45]
5-B	3D2C*	−	4D3 + M137P	347.25	−	−	-1.03	320.5	[45]
6B	3QMM	1.89	4D3 + M134E, M137P, S163P	351.35	−	−	-1.20	324.0	[47]

^[a]^ Names of *Bs*LipA structures are taken from the respective references.

^[b]^ A PDB ID marked with an asterisk indicates that the model of the corresponding variant was built using the structure with that PDB ID as a template.

^[c]^ In Å.

^[d]^ The temperature at which the unfolding transition begins.

^[e]^ Median stability of rigid contacts between residue neighbors computed by applying the ENT^FNC^ approach (see section “Median stability of rigid contacts between residue neighbors as a new measure for predicting thermodynamic thermostability”) (left column). Values in the right column were obtained by converting the median stabilities to a temperature scale according to Eq 1.

^[f]^ *T*_50_^60^ values, i.e., the temperature required to reduce the initial enzymatic activity by 50% within 60 min.

^[g]^ *T*_50_^15^ values, i.e., the temperature required to reduce the initial enzymatic activity by 50% within 15 min.

^[h]^ Average ${\tilde{rc}}_{ij,neighbor}$ over six wild type structures (see the main text for details).

### Construction of the constraint network and rigidity analysis

As described in the previous section, only the protein part was considered for network construction, i.e., all non-protein molecules including water molecules were discarded. This was done based on previous findings that including water molecules does not significantly change the rigidity analysis results [64, 65]. Proteins were modeled as constraint networks in a *body-and-bar* representation (see section “Body-and-bar networks” in S1 File) [66, 67] using the CNA software [35] that acts as a front- and back-end to the Floppy Inclusion and Rigid Substructure Topography (FIRST) program [51, 68]. Once the constraint network is built, rigidity analysis is carried out, which identifies (rigid) clusters of atoms with no internal motion and flexible links in between, using the pebble game algorithm [52, 53] as implemented in the FIRST software [51].

### Thermal unfolding simulation

By sequentially removing non-covalent constraints from a network, one can simulate a loss of structural rigidity due to a temperature rise. Specifically, hydrogen bonds were removed from the network in increasing order of their strength following the idea that stronger hydrogen bonds break at higher temperatures than weaker ones [69]. As such, only hydrogen bonds with an energy *E*_HB_ ≤ *E*_cut_(σ) were included in the network of state σ. A thermal unfolding trajectory of 60 network states was generated for each input network by decreasing *E*_cut_ from −0.1 kcal mol^−1^ to −6.0 kcal mol^−1^ with a step size of 0.1 kcal mol^−1^. According to the linear relationship between *E*_cut_ and the temperature *T* introduced by Radestock and Gohlke on 20 pairs of orthologs from mesophilic and thermophilic organisms, respectively (Eq 1) [12, 13], the range of *E*_cut_ used in this study is equivalent to increasing the temperature of the system from 302 K to 420 K with a step size of 2 K. Because hydrophobic interactions remain constant or become even stronger as the temperature increases [70, 71], the number of hydrophobic tethers were kept unchanged throughout the thermal unfolding simulation. Rigidity analysis was performed on all such generated network states, and then local and global rigidity characteristics were calculated (see section “Local and global rigidity indices” in S1 File). The setup of the thermal unfolding simulation and the subsequent rigidity analysis were performed using the CNA software [35], which is available from http://cpclab.uni-duesseldorf.de/software. A web service for performing CNA analysis can be accessed via http://cpclab.uni-duesseldorf.de/cna [34].

$$T=\frac{-20\;\text{K}}{{\text{kcal}*\text{mol}}^{-1}}E_{cut}+300\;\text{K}$$ (1)

### Ensemble of networks generated by using fuzzy noncovalent constraints

For improving the robustness of rigidity analyses, CNA is generally carried out on an ensemble of structures (e.g., generated by molecular dynamics (MD) simulations), and then results are averaged [11, 64]. The preceding MD simulation compromises the efficiency of the rigidity analysis, however. To overcome this drawback, Pfleger *et al*. [56] recently introduced an approach that performs rigidity analyses on an ensemble of network topologies (ENT^FNC^) generated from a single input structure by using fuzzy noncovalent constraints. Here, the number and distribution of non-covalent constraints (hydrogen bonds and hydrophobic tethers) are modulated by random components within certain ranges as specified in ref. [56], thus simulating thermal fluctuations of a biomacromolecule without actually moving atoms. An ensemble of 2000 network configurations was generated using these definitions of fuzzy noncovalent constraints for all *Bs*LipA variants, respectively. Finally, average local indices were calculated, as were average phase transition temperatures identified by the global index cluster configuration entropy *H*_type2_. The index *H*_type2_ monitors the degree of disorder in the realization of a given network state σ: As long as a network is dominated by a very large rigid cluster, *H*_type2_ tends to be low because there are only a few configurations of a system with a large rigid cluster possible; *H*_type2_ increases when larger rigid clusters break down in smaller clusters (see section “Local and global rigidity indices” in S1 File and ref. [55] for details).

### Clustering of unfolding pathways

Recently, we showed that curves of the rigidity order parameter, which characterizes the general percolation behavior of a constraint network during thermal unfolding, for mesophilic proteins and their thermophilic counterparts are almost identical except for a shift of the curve of the thermophilic protein to higher temperatures [12]. This finding supported the hypothesis of corresponding states according to which mesophilic and thermophilic enzymes are in corresponding states of similar rigidity and flexibility at their respective optimal temperature [12]. The percolation index *p*_i_ is a local analog to the rigidity order parameter. It monitors for each bond when it segregates from the largest rigid cluster present at the beginning of a thermal unfolding simulation (see section “Local and global rigidity indices” in S1 File and ref. [55] for details). That way, a residue-wise *p*_i_ profile of a protein, generated by taking the lower of the *p*_i_ values of the two backbone bonds for each residue, expresses the hierarchical break-down of the largest rigid cluster during a thermal unfolding simulation.

**Fig 1:**
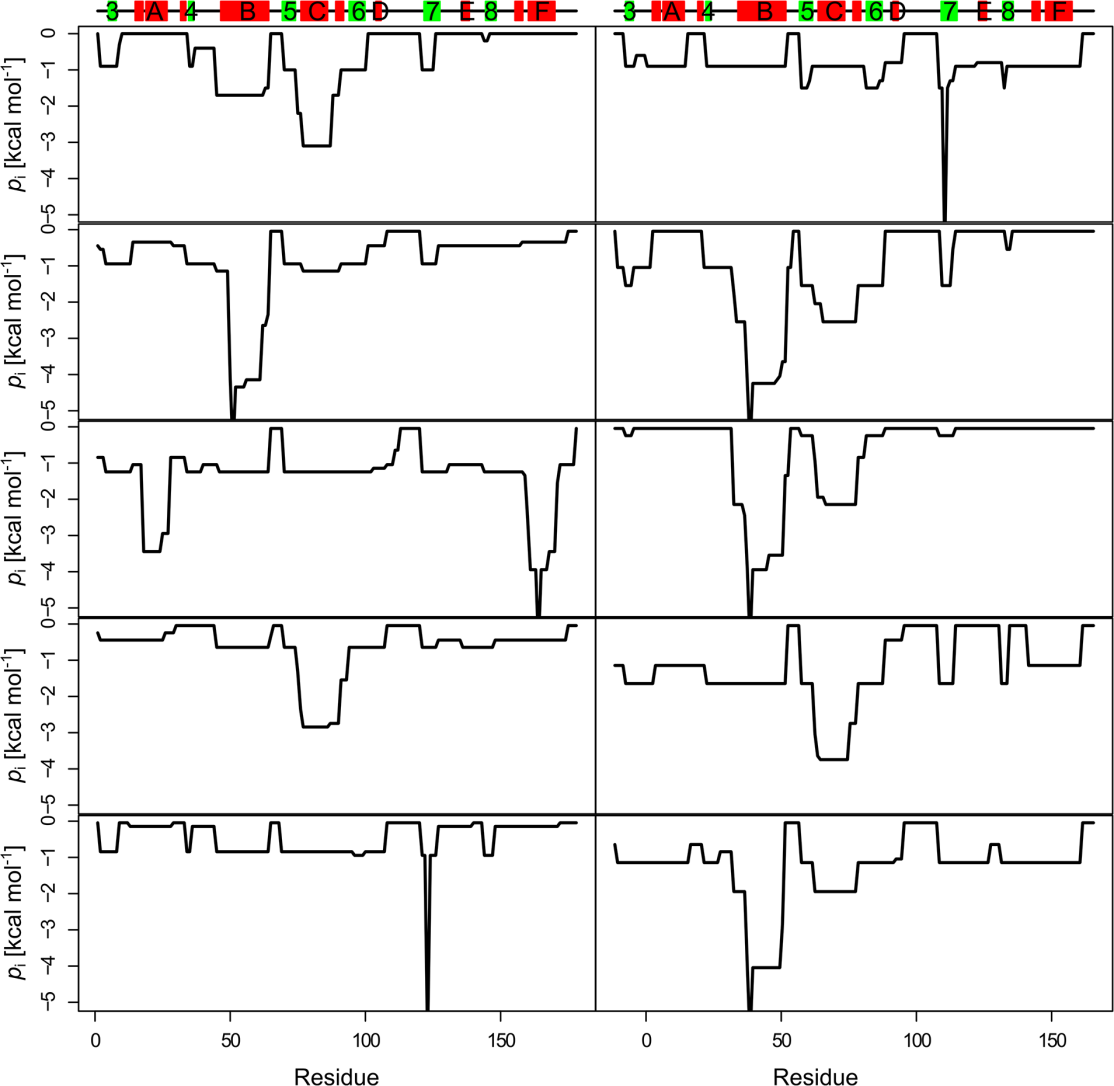
Residue-wise *p*_i_ plots for medoids of the 10 clusters. Secondary structure elements as computed by the DSSP program [88, 89] are indicated on the top of the plots and are labeled: α-helix (red rectangle), β-strands (green rectangle), loop (black line).

We thus reasoned that the (dis)similarity of unfolding pathways of *Bs*LipA variants can be measured by Manhattan distances between their respective *p*_i_ profiles. We used this distance measure for clustering the network topologies of all *Bs*LipA variants into 10 clusters using the Partitioning Around Medoids algorithm [72] as implemented in the R program (http://www.r-project.org). This optimal number of clusters was chosen based on monitoring the change in the objective function of the clustering (the mean of the dissimilarities of all objects to their nearest medoids) as a function of the number of clusters (Figure A in S1 File) and visual inspection of cluster medoids for their dissimilarity to other medoids (residue-wise *p*_i_ profiles for medoids of the 10 clusters are shown in Fig 1). A clustering in more than 10 clusters essentially created additional clusters that were very similar to other clusters. From this, the cluster distribution (frequencies of network topologies in each of the 10 clusters out of in total 2000 network topologies) for each *Bs*LipA variant was calculated by counting the number of networks that belongs to each of the 10 clusters. A high (low) correlation between cluster distributions for two *Bs*LipA variants then indicates that both variants unfold in a similar (different) manner. Finally, a matrix of all pairwise correlations of cluster distributions of *Bs*LipA variants was generated.

## Results

### Data set

**Fig 2:**
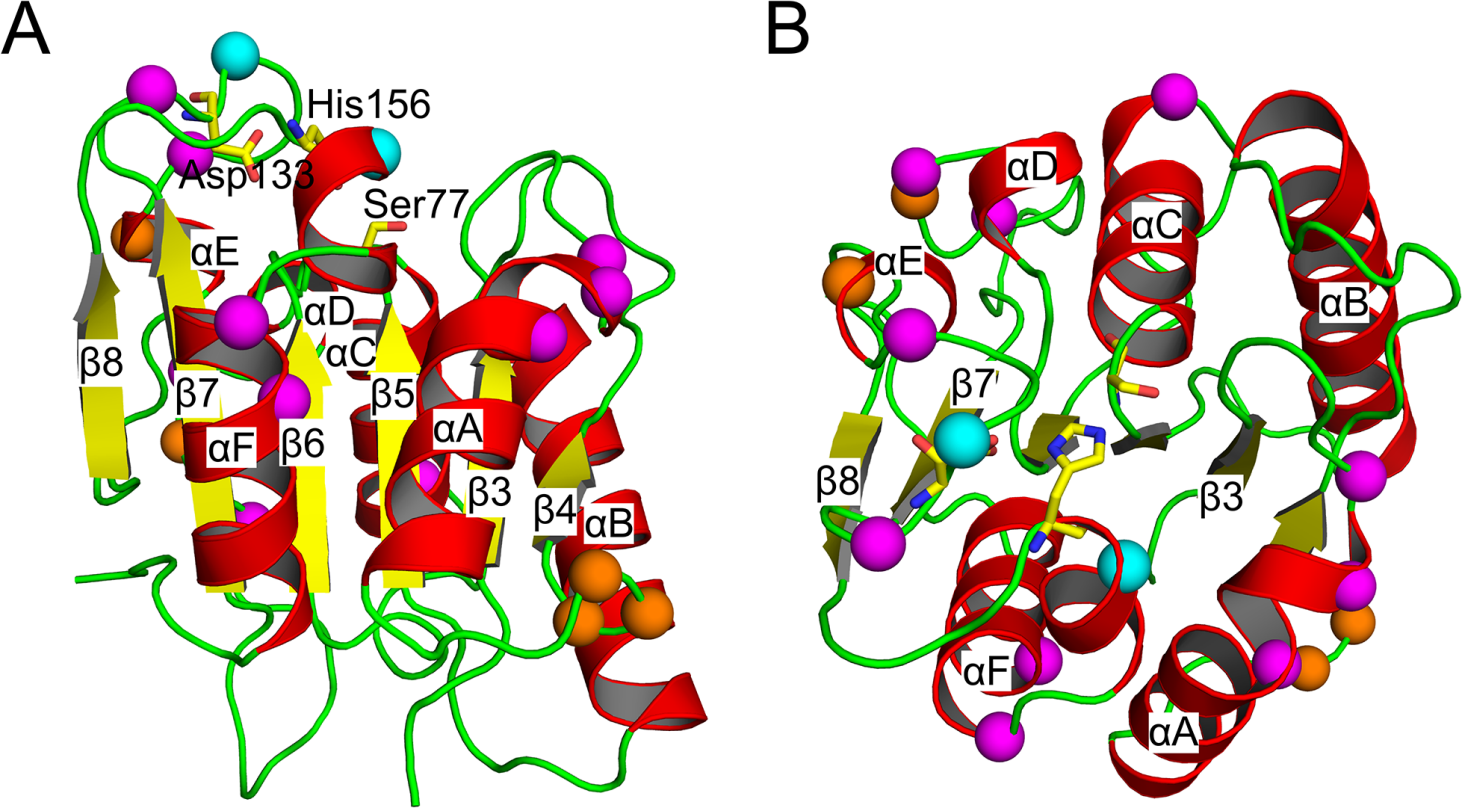
Cartoon representation of wild type *Bs*LipA with mutated residues indicated by spheres of their C_α_ atoms (mutations from Rao *et al*. [44–48]: magenta; Reetz *et al*. [42, 49]: orange; mutations common in both data sets: cyan). The catalytic triad (Ser77-Asp133-His156) is shown in stick representation with yellow carbons. The protein is colored according to secondary structure (α-helices: red; β-sheets: yellow; loops: green). The right view (**B**) differs from the left (**A**) by an anti-clockwise rotation of ~90° about a horizontal axis. All figures of *Bs*LipA structures were generated with PyMOL (http://www.pymol.org).

*Bs*LipA is a protein of 181 amino acids with a minimal α/β hydrolase fold; in this fold, a central parallel β-sheet of six β-strands is surrounded by six α-helices. Ser77, Asp133, and His156 constitute the catalytic triad (Fig 2). Unlike other lipases, the catalytic site in *Bs*LipA is not covered with a lid. Hence, *Bs*LipA does not show interfacial activation [40]. The data set used in this study contains structures of the wild type *Bs*LipA, thirteen mutants from Rao *et al*. [44–48], and three mutants from Reetz *et al*. [42, 49] (Table 1). The mutants differ from the wild type by three to twelve mutations, i.e., the sequence identity is > 93%. Models for the mutants for which X-ray structures were not available were built using the SCWRL program. As the number of mutations in the modeled variants is ≤ 7 with respect to the template structures (< 4% with respect to the sequence length) (Table 1), an overall similar backbone confirmation can be expected as can be an overall reliable modeling of side chain conformations by SCWRL. This was also evident from a very good structural alignment and low root-mean-square deviations (RMSD) between the wild type and those mutants for which crystal structures were available (C_α_ atom-based RMSD values between the wild type and the mutants < 0.38 Å). The high structural similarity allows a direct comparison of results from rigidity analyses for these structures [11–13].

The melting temperature *T*_m_ of the wild type is 329.15 K. The *T*_m_ values of the mutants of Rao *et al*. range from 334.35 to 351.35 K (Table 1). For the mutants of Reetz *et al*. no *T*_m_ values are available. Rather, unfolding initiation temperatures *T*_i_ were reported, which are lower by 2.5 to 6.2 K than that of the wild type. This suggests that mutants of Reetz *et al*. are thermodynamically less thermostable than the wild type [49], in contrast to mutants from Rao *et al*. [44–48]. However, we note that, while *T*_m_ reports on the temperature at which 50% of the protein is unfolded and, hence, properly describes the folded-unfolded equilibrium of a protein, *T*_i_ only reports on the temperature at which the unfolding transition begins. Therefore, we will only consider relations within mutants of Rao *et al*. and to the wild type and distinguish those from relations within mutants of Reetz *et al*. and to the wild type. Finally, the *T*_50_^*t*^ values of the mutants of Reetz *et al*. are higher than that of the wild type (Table 1), showing that these mutants more efficiently refold upon cooling after incubation at high temperatures than does the wild type. The location of mutations in all of the mutants investigated in this study is shown in Fig 2; all mutations are located on the protein surface.

### Thermal unfolding pathway of *Bs*LipA

**Fig 3:**
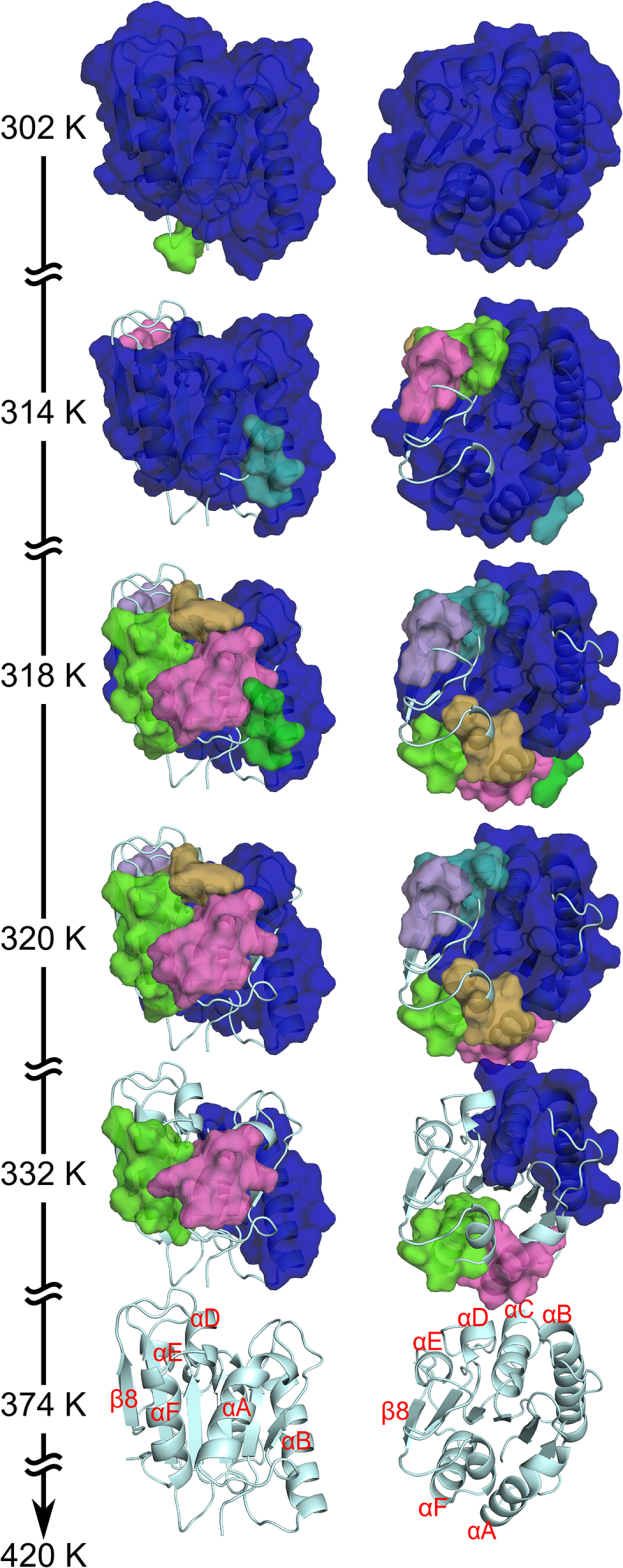
Average loss of structural rigidity of the wild type *Bs*LipA during a thermal unfolding simulation. Rigid clusters are depicted as uniformly colored bodies, with the largest rigid cluster shown in blue and smaller rigid clusters in the order of the colors green, magenta, cyan, orange, and violet. Temperatures are indicated for each depiction of a rigid cluster decomposition. At the beginning of the thermal unfolding simulation (302 K), almost the complete structure is part of the giant rigid cluster; in contrast, the structure becomes completely flexible at temperatures ≥374 K. The right views differ from the left ones by an anti-clockwise rotation of ~90° about a horizontal axis. Important secondary structure elements are labeled. Note that the unfolding pathway shown here represents an *average loss of rigidity percolation* calculated from a stability map (see section “Local and global rigidity indices” in S1 File) averaged over all unfolding trajectories obtained for the ensemble of 2000 network topologies. Hence, the temperature at the phase transition point identified that way (Figure B in S1 File) cannot be compared to the *average phase transition temperature*, which is obtained from 2000 individual *T*_**p**_ values and used for predicting the thermodynamic thermostability of *Bs*LipA variants (see section “Prediction of thermodynamic thermostability of *BsLipA* variants”)

From monitoring the loss in rigidity percolation during thermal unfolding simulations, major phase transitions in the protein can be identified that relate to the unfolding pathway [11–13, 54, 73]. Here, we describe the loss of rigidity percolation of the wild type *Bs*LipA (PDB ID 1ISP) as an example. Similarity or dissimilarity, respectively, of the unfolding pathways across all variants is described below. During the thermal unfolding, a giant rigid cluster that exists at low temperature (equivalent to a high *E*_cut_) breaks down in smaller sub-clusters until, finally, the whole protein becomes flexible at a high temperature (Fig 3; see also S1 Video showing the loss of rigidity percolation during the thermal unfolding of the wild type). As such, nearly the entire protein structure constitutes a single giant rigid cluster initially (at 302 K; Fig 3). As the temperature increases, loops segregate first from the giant rigid cluster. Then, at 314 K, α-helix D (αD) and αE segregate to form individual small rigid clusters (Fig 3), as do αA and αF at 318 K. The giant rigid cluster at this temperature is formed by the central β-sheet region and the two helices αB and αC (Fig 3). Next, the β-sheet region becomes sequentially flexible, beginning with β4 and β8 at 320 K (Fig 3). Then, the remaining β-strands become flexible in the order β3, β7, and β5−β6, leading to a completely flexible β-sheet region at 332 K (Fig 3 The immediate next step at which αB and αC become two separate rigid clusters is identified as a phase transition point: Now most of the structure has become flexible. This transition is most prominent with respect to going from a structurally stable wild type *Bs*LipA to an unfolded one (Figure B in S1 File). After this phase transition point, the remaining rigidity is sequentially lost, and the structure finally becomes completely flexible at 374 K (Fig 3).

During the thermal unfolding of *Bs*LipA, helices segregate from the giant rigid cluster as independent small rigid clusters. This is due to two reasons: First, in the *body-and-bar* network representation, a helix with a minimum of seven amino acids is already rigid by itself due to constraints arising from covalent and backbone hydrogen bonds [66]. Second, with the current energy function *E*_HB_ [69], all backbone hydrogen bonds are assigned a very similar strength, irrespective of their location along a helix. Thus, a helix will persist as an independent rigid cluster during the thermal unfolding simulation until all backbone hydrogen bonds break almost simultaneously at a high temperature, which most likely represents an overstabilization of a helix [74]. Considering this behavior, the unfolding pathway identified for the wild type *Bs*LipA is in good agreement with respect to the early segregation of α-helices with experimental findings on the unfolding of proteins with an α/β hydrolase fold [75, 76]. This indicates that side chain-mediated interactions between amino acids are well represented by the applied definitions of non-covalent constraints in the network. This is important as we want to detect effects of changes in such interactions due to mutations.

### Prediction of thermodynamic thermostability of *Bs*LipA variants based on the global index *H*_type2_

**Fig 4:**
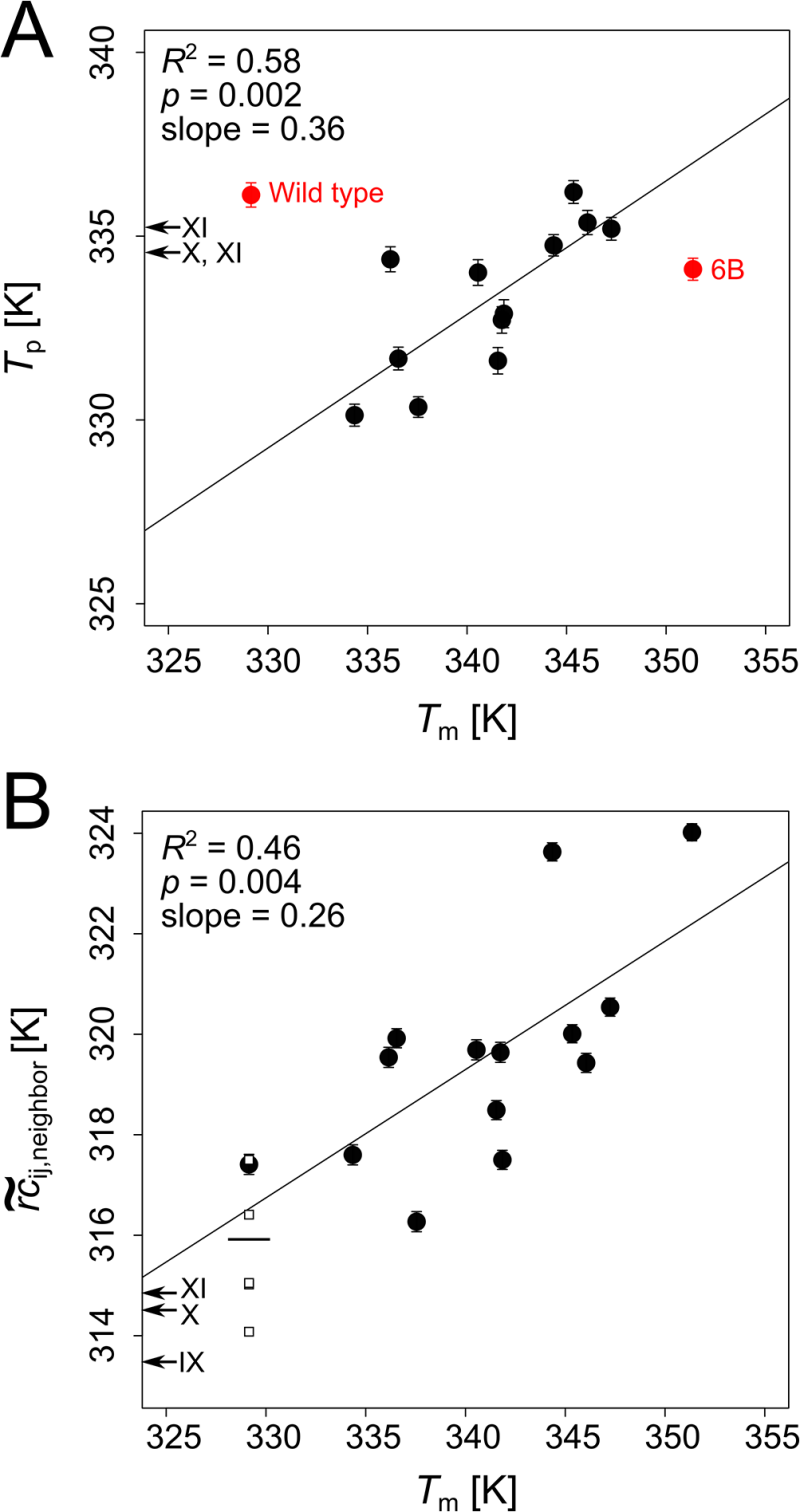
Correlation between predicted and experimental thermostabilities (*T*_m_ values) of *Bs*LipA variants; for the predictions, the ENT^FNC^ approach was used. **A**: Correlation between *T*_**p**_ derived from the global index *H*_**type2**_ and *T*_**m**_ values for thermodynamically thermostabilized mutants from Rao *et al*. Data points colored red were considered outliers (see main text for explanation) and excluded when calculating *R* ^**2**^ values and the correlation lines. **B**: Correlation between ${\tilde{rc}}_{ij,neighbor}$ and *T*_**m**_ values for thermodynamically thermostabilized mutants from Rao *et al*. Data points shown as empty squares represent ${\tilde{rc}}_{ij,neighbor}$ values for five additional wild type crystal structures (see main text for details; two of the squares closely overlap; mean ${\tilde{rc}}_{ij,neighbor}$ over all six data points for wild type structures is shown as a small horizontal line: 315.9 ± 0.6 K). A and B: Error bars represent the standard error in the mean. *T*_**p**_ and ${\tilde{rc}}_{ij,neighbor}$ values for kinetically thermostabilized mutants from Reetz *et al*. are marked by arrows on the corresponding ordinates.

From the thermal unfolding simulations, the temperature of the phase transition point *T*_p_ was identified as described in the section “Local and global rigidity indices” in S1 File. Note that *T*_p_ values determined that way should be considered relative values only, as stated in previous studies [12, 34, 35]. Initially, we calculated phase transition points using single network topologies generated from the input structures of wild type *Bs*LipA and mutants of Rao *et al*.; however, this resulted in a very poor prediction of thermodynamic thermostability with a coefficient of determination (*R* ^2^) for a linear fit between experimental *T*_m_ and predicted *T*_p_ of 0.22 (Figure C in S1 File). We anticipated that this result reflects the high sensitivity of CNA on the conformation of the input structures as also found previously [11, 56, 64, 65]. We thus resorted to averaging *T*_p_ values over an ensemble of *Bs*LipA, applying the recently developed ENT^FNC^ approach. This approach generates an ensemble of network topologies from a single input structure and has been shown to yield results of rigidity analyses both at the local and global level that agree almost perfectly with those obtained from MD simulations-generated ensembles of structures [56]. However, this yielded a significant (*p* = 0.002) correlation between *T*_p_ and *T*_m_ with *R* ^2^ = 0.58 only if the two structures with the lowest (wild type) and highest (mutant 6B) *T*_m_ were considered outliers (Fig 4A; see below for an explanation regarding the outliers; note that removing the two outliers in the case of using single network topologies only marginally improved *R* ^2^ from 0.22 to 0.29). The mutants IX, X and XI of Reetz *et al*. were predicted to be slightly less thermostable than the wild type (Fig 4A). This is in line with experimental findings by Reetz *et al*. that suggest that these mutants are thermodynamically less stable than the wild type [49]. In summary, these results suggest that CNA coupled with the ENT^FNC^ approach can sense effects on the thermodynamic thermostability that arise from only a few sequence variations (pairwise sequence identity > 93%; pairwise RMSD < 0.38 Å). However, the false predictions for wild type *Bs*LipA and mutant 6B are dissatisfying.

### Difference in unfolding pathways explains outliers

**Fig 5:**
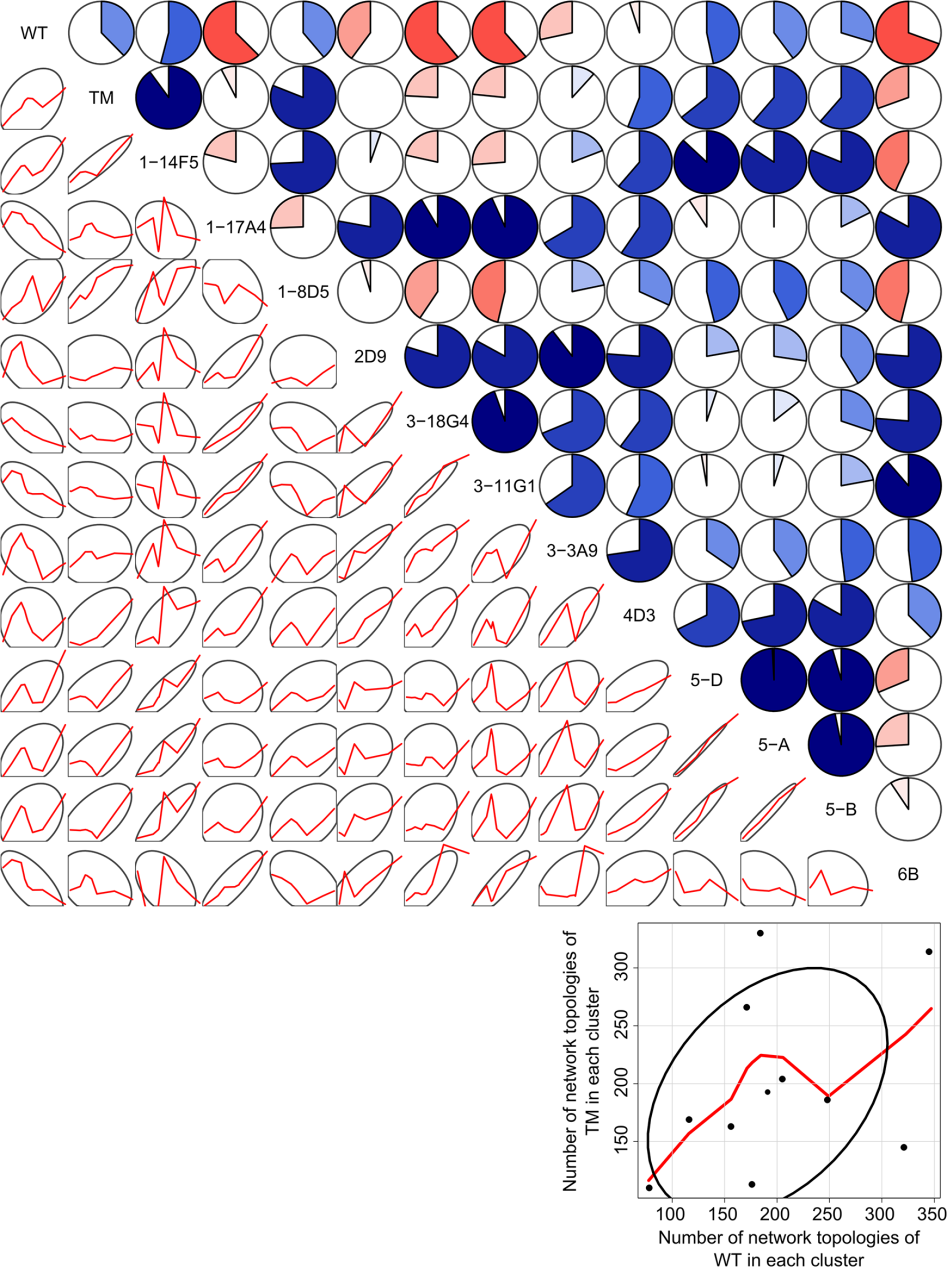
Pairwise correlations of cluster distributions (using 10 clusters) of unfolding pathways of wild type *Bs*LipA and mutants from Rao *et al*. The upper triangle shows pairwise correlation coefficients as dial plots where a filled portion of a pie indicates the magnitude of the correlation (*r*) and blue (red) color indicates a positive (negative) correlation. The lower triangle shows 68% data ellipses (depicting the bivariate mean ± 1 standard deviation) [90] and scatterplots of the respective cluster distributions (frequencies of network topologies in each of the 10 clusters) of the two *Bs*LipA variants as red lines smoothed by locally-weighted polynomial regression [91]. Data points and axes for the plots in the lower triangle are omitted for clarity. The blow-up in the right bottom corner depicts exemplarily for the TM *versus* WT case axes, axes labels, data points, the smoothing line obtained by locally-weighted polynomial regression, and the 68% data ellipsis. The figure was generated using the “corrgram” package [92] of the R program (http://www.r-project.org).

Next, we investigated why the thermostabilities of the wild type and the mutant 6B were predicted falsely. Since the precision of the computations shown in Fig 4A is high (the standard error in the mean is < 0.38 K in all cases), we reasoned that the false prediction must arise from a systematic difference between the wild type and 6B *versus* all other mutants of Rao *et al*. Thus, we mutually compared all unfolding pathways of the systems as described in “Materials and Methods”. After partitioning unfolding pathways of *Bs*LipA variants characterized on a residue basis by the percolation index *p*_i_ into 10 clusters (see Fig 1 for the *p*_i_ profiles of the 10 cluster medoids), we calculated correlation coefficients from the resulting cluster distributions for all pairs of variants (Fig 5; Tables A and B in S1 File).

These results revealed that the wild type enzyme shows an unfolding pathway distribution very distinct from other *Bs*LipA variants from Rao *et al*. with correlation coefficients *r* ranging from −0.69 to 0.54 (Fig 5, Table A in S1 File). The average *r* value for the wild type against all other variants from Rao *et al*. is −0.06 ± 0.14 (mean ± SEM), which is lower than that of the other variants (≥°0.16 except for the outlier 6B) (Table A in S1 File). The second outlier, mutant 6B, has an average *r* value of 0.12 ± 0.16 when comparing its unfolding pathway distribution to those of other variants from Rao *et al*. This average *r* value is lower than the corresponding average *r* values of all other mutants from Rao *et al*. (Table A in S1 File). The thermal unfolding pathway of 6B is shown in Figure D in S1 File. While the overall unfolding pathway of 6B is comparable with that of the wild type *Bs*LipA in that the helices segregate from the giant rigid cluster as individual rigid clusters in the early phase of unfolding, they do so in a different order (αD, αA−αF, αE, αB−αC; Figure D in S1 File). A probability density function (PDF) of *r* values of unfolding pathway distributions of the two outliers wild type and mutant 6B with all other variants shows a bimodal distribution and is shifted towards lower *r* values compared to the PDF of the *r* values of other mutants from Rao *et al*. Furthermore, about half of this distribution is related to negative *r* values (Figure E in S1 File). In all, this suggests that the two outliers have unfolding pathways different from all other mutants from Rao *et al*. for which the prediction of thermodynamic thermostability was successful. Finally, we note that the unfolding pathway distributions of the wild type and the three mutants from Reetz *et al*. are highly similar to each other (*r* > 0.79; *p* ≤ 0.001; Table B in S1 File).

These findings have important implications: First, the results strongly suggest that the misprediction of the thermostabilities of the wild type and mutant 6B arises from them showing different unfolding pathways from all of the remaining mutants from Rao *et al*.. Apparently, the present approach of identifying phase transition points by monitoring the *global* index *H*_type2_ (see section “Local and global rigidity indices” in S1 File) is too sensitive with respect to the details of such pathways. Consequently, alternative methods should be explored (see section “Median stability of rigid contacts between residue neighbors as a new measure for predicting thermodynamic thermostability”). Second, the results suggest that the history of the generation of the *Bs*LipA structures may play a role for the observed differences in the unfolding pathways: generally, the most similar unfolding pathways (Tables A and B in S1 File) (and then the most coherent *T*_p_ predictions) are found for those variants that originate from a common structural “ancestor” (Table 1). Third, the results propose to apply the similarity/dissimilarity of unfolding pathway distributions as a measure to judge the reliability of thermostability predictions in future studies: the lower the similarity for two variants, the less confident should one be that relative thermostability predictions are correct. Finally, we cannot exclude at the present stage that thermostabilizing mutations lead to an unfolding pathway that is different from the one of the wild type. Considering that intrinsic and extrinsic modifications in other systems that led to thermostabilization have been shown to influence not just the folded state but the entire (un)folding free energy landscape [77, 78], this possibility also exists for *Bs*LipA mutants [45, 47].

### Median stability of rigid contacts between residue neighbors as a new measure for predicting thermodynamic thermostability

The above findings called for predicting the thermodynamic thermostability in a way that is less sensitive to the details of the unfolding pathway than the present approach relying on the *global* index *H*_type2_. The sensitivity arises here from the need to accurately identify the phase transition point from the percolation behavior of the constraint network as the most pronounced jump in *H*_type2_ during the unfolding (Figure B in S1 File). As shown previously, however, the percolation behavior of networks from protein structures is complex [13] (in contrast to that of network glasses [54, 79]), reflecting that a protein structure is hierarchical and composed of modules. As a consequence, often more than one pronounced jump in *H*_type2_ is observed, which then makes it difficult to assign a phase transition point (Figure B in S1 File).

As an alternative, we set out to characterize thermodynamic thermostability at the *local* level [55], i.e., by monitoring residue pair-wise descriptors of local stability within a protein structure as a function of the temperature. The most comprehensive information in that direction is provided by stability maps *rc*_*ij*_ [12], which depict when a rigid contact *rc* between two residues *i* and *j* ceases to exist along a thermal unfolding trajectory. As such, *rc*_*ij*_ contains information cumulated over all states *σ* of a network along the trajectory as to which parts of the network are (locally) mechanically stable at a given state *σ*, and which are not [12, 55]. Of note, this stability information is not only available in a qualitative manner (i.e., in terms of local rigidity and flexibility) but also quantitatively in that each *rc*_*ij*_ has associated with it the energy *E*_cut_ at which this rigid contact is lost. Thus, ∑_*i*,*j* > *i*_ *rc*_*ij*_ represents the chemical potential energy due to non-covalent bonding, obtained from the coarse-grained, residue-wise network representation of the underlying protein structure. With respect to a reference state where no non-covalent interactions are present anymore (i.e., an unfolded state), ∑_*i*,*j* > *i*_ *rc*_*ij*_ can be considered an unfolding energy then. Three modifications were applied to ∑_*i*,*j* > *i*_ *rc*_*ij*_ here for technical reasons. I) In order to stress the locality of interactions within a protein, which will later aid in understanding how structural differences relate to thermostability differences (see section “Influence of mutations on local structural rigidity”), we focused on the stability of rigid contacts *rc*_*ij*,*neighbor*_ between structurally close residues only (i.e., those residues where at least one pair of respective atoms is within 5 Å distance). II) To suppress the influence of extreme values in the double summation on the outcome of the unfolding energy, we used the median stability of rigid contacts ${\tilde{rc}}_{ij,neighbor}$ instead. Such extreme values can occur in regions that are highly stabilized by interactions to hydrophobic atoms [56]. III) Applying the ENT^FNC^ approach, ${\tilde{rc}}_{ij,neighbor}$ were averaged over ensembles of 2000 constraint networks, which has been shown to significantly improve the robustness of rigidity analyses [56]. The ${\tilde{rc}}_{ij,neighbor}$ values are given in Table 1. In addition, Table 1 and Fig 4B show these values after converting them to a temperature scale via Eq 1

A significant and fair linear correlation of ${\tilde{rc}}_{ij,neighbor}$ with *T*_m_ values of the thermodynamically stable mutants from Rao *et al*. is obtained (*R* ^2^ = 0.46, *p* = 0.004; Fig 4B). No outlier is observed now, indicating that our definition of an average local stability correctly reflects differences in the thermodynamic thermostability. This finding substantiates our above interpretation of ${\tilde{rc}}_{ij,neighbor}$ as an approximation to the unfolding energy, because under the condition of a temperature-independent heat capacity the unfolding energy is linearly correlated to the melting temperature, with the heat capacity as the scaling factor [80]. The slope of the correlation line (0.26) in Fig 4B deviates from unity. This indicates that the linear relationship in Eq 1 used for converting ${\tilde{rc}}_{ij,neighbor}$ to a temperature scale, which was derived for *H*_type2_-based thermostability prediction [12, 13], may need to be reparameterized for application with ${\tilde{rc}}_{ij,neighbor}$. In this case, as the heat capacity has been shown to scale linearly with the number of residues for small globular proteins [80, 81], a normalization with respect to protein size needs to be applied. When only considering the six X-ray structures in the dataset of Rao *et al*., a good correlation of ${\tilde{rc}}_{ij,neighbor}$ with *T*_m_ values of *R* ^2^ = 0.87 (*p* = 0.007) is found (Table 1). In contrast, a weaker correlation (*R* ^2^ = 0.33, *p* = 0.07) is obtained for the eight variants that were modeled using SCWRL (). This suggests an influence of the quality of the input structures on the prediction of thermodynamic thermostability. Finally, as before, the mutants from Reetz *et al*. are found to have a lower thermodynamic thermostability than the wild type, in very good agreement with experimental findings (see above and Table 1) [49].

The ${\tilde{rc}}_{ij,neighbor}$-based measure is apparently less sensitive to differences in the unfolding pathway because the wild type and mutant 6B are now much better ranked. However, comparing the prediction of thermostabilities by ${\tilde{rc}}_{ij,neighbor}$ and *H*_type2_, the latter yields a better correlation with *T*_m_ for mutants with similar unfolding pathways. From an application point of view, we thus recommend using *H*_type2_-derived *T*_p_ values for comparing thermostabilities of variants of a protein unless the underlying unfolding pathways are dissimilar; in that case, we recommend using ${\tilde{rc}}_{ij,neighbor}$.

When applied to hen egg white lysozyme the ENT^FNC^ approach has been shown to significantly improve the robustness of rigidity analyses with respect to the conformation of the input structures [56]. To probe if this also holds for *Bs*LipA investigated here, we computed ${\tilde{rc}}_{ij,neighbor}$ using the ENT^FNC^ approach for five additional crystal structures of wild type *Bs*LipA (see section “Materials and methods”). The standard error of the mean in ${\tilde{rc}}_{ij,neighbor}$ over all six wild type *Bs*LipA structures is 0.57 K (Fig 4B) including PDB ID 1ISP discussed so far. This error is likely within the experimental uncertainty, confirming our previous results of robust rigidity analyses with ENT^FNC^ [56]. Still, if the average ${\tilde{rc}}_{ij,neighbor}$ over all six crystal structures (315.9 K; see horizontal line in Fig 4B;Table 1) is considered for the ${\tilde{rc}}_{ij,neighbor}$ *versus T*_m_ correlation, the quality of the correlation improves considerably to *R* ^2^ = 0.55 (*p* = 0.001) compared to if only ${\tilde{rc}}_{ij,neighbor}$ of PDB ID 1ISP is used (see above). This indicates that the use of multiple input structures in connection with the ENT^FNC^ approach further increases the accuracy of thermostability predictions.

### Influence of mutations on local structural rigidity

**Fig 6:**
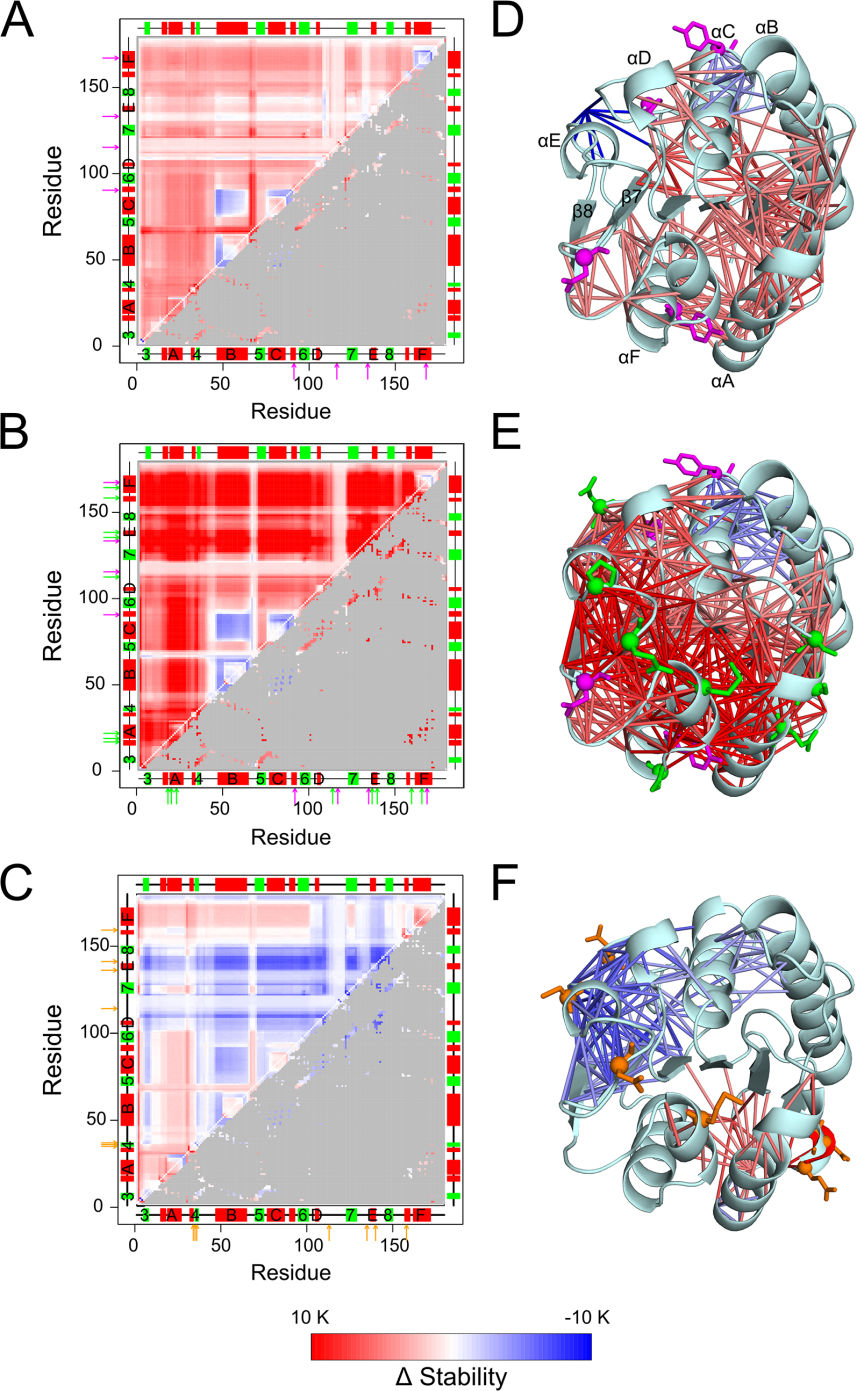
Differences in the stability of rigid contacts between wild type and mutants of *Bs*LipA. Maps depict differences between stability maps of the respective mutants and an average stability map of the six wild type structures (see the main text for explanation) for **A**: mutant 1-14F5, **B**: mutant 6B, and **C**: mutant X. A red (blue) color indicates that a rigid contact in the mutant is more (less) stable than in the wild type (see color scale at the bottom). The upper triangles show differences in the stability values for all residue pairs; the lower triangles show differences in the stability values only for residue pairs that are within 5 Å of each other, with values for all other residue pairs colored gray. Secondary structure elements as computed by the DSSP program [88, 89] are indicated on both abscissa and ordinate and are labeled: α-helix (red rectangle), β-strands (green rectangle), loop (black line). Arrows represent the mutation positions with respect to the wild type sequence: Common mutations in 1-14F5 (A) and 6B (B) are shown in magenta, unique mutations in 6B (B) are shown in green, and mutations in X (C) are shown in orange. The differences in the stability of rigid contacts for residue neighbors is also displayed on the structures of the mutants by sticks connecting C_**α**_ atoms of residue pairs colored according to the color scale of the maps for **D**: 1-14F5, **E**: 6B, and **F**: X. Only those contacts that are stabilized by ≥ 4 K or destabilized by ≥ 3 K are shown for clarity; for the same reason, contacts between two residues of the same secondary structure element are not shown. Mutated residues are shown as sticks and a sphere at their C_**α**_ atoms (D, E, and F) in the same color used for arrows (A, B, and C).

Considering that the average local stability defined above correctly reflects differences in the (macroscopic) thermodynamic thermostability, we analyzed on a residue basis how changes in thermostability relate to changes in local structural stability (rigidity). First, we compared stability maps of variants from Rao *et al*. with distinct thermostabilities to analyze the effect of mutations on the local rigidity. In particular, we compared the wild type to a more thermostable variant 1-14F5 and the most thermostable variant 6B. We averaged stability maps of the six wild type structures (see above and Fig 4B) and used this average for comparison against the thermostable variants of *Bs*LipA. Difference stability maps for 1-14F5/wild type (Fig 6A) and 6B/wild type (Fig 6B) pairs demonstrate that mutations in general improve the strength of rigid contacts to and in between neighboring residues of the mutations (lower triangles in Fig 6A and 6B) but also in between residue pairs not in contact distance (upper triangles in Fig 6A and 6B). This effect is more pronounced for 6B/wild type than 1-14F5/wild type.

In more detail, the four mutations (indicated by arrows in Fig 6A and shown in Fig 6D) on 1-14F5 stabilize contacts of αD with its neighboring helix αC and contacts of αA with αF (Fig 6A and 6D). More importantly, the contacts of helices αA and αF with their neighboring β-strands in the central β-sheet region are stabilized, which delays the early loss of these helices observed during the thermal unfolding of the wild type (Fig 3). Similarly, the contacts between αB and the central β-sheet region also become stronger, which delays the decay of structural stability of the β-sheet during thermal unfolding. On average, contacts between all residue neighbors are ~−0.1 kcal mol^−1^ or ~2 K more stable in 1-14F5 than in the wild type.

Residues mutated in 6B (indicated by arrows in Fig 6B and shown in Fig 6E) include the mutations already found in 1-14F5. This explains a strengthening of inter-helical contacts and of the contacts between α helices and the central β-sheet region as discussed already for 1-14F5 (Fig 6D and 6E). However, the additional mutations in 6B stabilize contacts between other α-helices (αD and αE) and the central β-sheet region and further reinforce those between αA or αF and the β-sheet. On average, contacts between all residue neighbors are ~−0.4 kcal mol^−1^ or ~8 K more stable in 6B than in the wild type (Fig 6E).

Taken together, contacts between peripheral helices and the central β-sheet region are stronger in 6B than in 1-14F5. This delays the loss of α-helices during thermal unfolding (Fig 3) to a larger extent in 6B than in 1-14F5, explaining at a structural level why 6B is more stable than 1-14F5. Remarkably, many of these stabilizations must arise from the long-range aspect of rigidity percolation [52, 64, 82, 83], because almost all mutations in 6B are on the surface, i.e., far from the central β-sheet region. In contrast, inter-helical contacts of the αB/αC helix pair become weaker in the mutants than in wild type (Fig 6D and 6E) indicating that the strengthened stability between these helices and the central β-sheet region is sufficient to keep the structure folded. At last, for all other thermodynamically more thermostable mutants, a similar profile of changes in contact stability between various secondary structure elements was observed (Figure F in S1 File). Not unexpected, the increase in contact stability compared to wild type (Figure F in S1 File) was generally the more pronounced the higher the thermodynamic thermostability is of the mutant (Table 1).

Second, we compared the mutants from Reetz *et al*. to the wild type. Regarding mutant X, seven residues have been mutated (indicated by arrows in Fig 6C and shown in Fig 6G). In strict contrast to what was observed for the thermodynamically thermostabilized mutants, this mutant showed a destabilization of rigid contacts both locally and globally (Fig 6C and 6F; see also Figure G in S1 File, where a similar finding is depicted for mutants IX and XI). For mutant X, the average decrease in stability over all residue neighbors is ~0.06 kcal mol^-1^ or ~1.2 K. The destabilization found on the local scale agrees with results of a lower *T*_p_ found when analyzing the mutants globally. Furthermore, the results are in line with experimental findings which suggest that the mutants are thermodynamically less stable than the wild type (Table 1) [49]. Our findings are also in good agreement with results obtained by comparative crystal structure analysis of wild type and variant X [49]: Loop region 14–21, for which lower B-factors in X than in the wild type structure were observed, shows increased contact stabilities with its neighboring residues in X (Fig 6C and 6F; Figure H in the S1 File). Likewise, regions 129–153 and 177–181, for which higher B-factors in X than in the wild type structure were observed, show decreased contact stabilities with their neighboring residues in X (Fig 6C and 6F; Figure H in the S1 File). However, region 60–70 shows increased contact stabilities in X (Fig 6C and 6F and Figure H in the S1 File) despite higher B-factors observed in the comparative crystal structure analysis. The latter may reflect increased motions of a stabilized region as a whole, taking into consideration that B-factors can report on rigid body motions of a structurally stable part [84].

Finally, it would be very satisfying from both the biochemical and structural biology point of view, if the effects of the three to twelve mutations on increased or decreased local rigidity could be immediately related to the changes in specific interactions with neighboring residues. Our above observation for the mutants from Rao *et al*. that many of the stability changes must arise from the long-range aspect of rigidity percolation [52, 64, 82, 83] speaks against such an endeavor. Nevertheless, we analyzed differences in the per-residue number of hydrogen bonds and hydrophobic tethers of the variants 1_14F5, 6B, and X with respect to the wild type (Figure I in S1 File). While these analyses reveal differences in the number of interactions > 1 indeed only for a small set of residues, between ~10 and ~35% of all residues show differences of 1. This finding is remarkable given the small number of mutations in the variants and the high structural similarity between crystal structures of the wild type and mutants found for backbone atoms above; apparently, these differences arise from subtle changes in the conformations of the side chains due to the mutations. This finding also suggests that an interpretation of the relationship between increased or decreased local rigidity due to changes in specific interactions of a mutated residue and a change in thermostability of a mutant may fall short of the actual complexity underlying this relationship.

## Discussion

Understanding the origin of thermostability is of fundamental importance in protein biochemistry. Here, we have probed the relation between protein thermostability and structural rigidity by directly analyzing static properties of a well-characterized set of 16 *Bs*LipA mutants. The main outcome of this work is the finding of a good correlation between the structural rigidity of all *Bs*LipA variants and their thermodynamic thermostability. This finding of a quantitative relation between structural rigidity and thermodynamic thermostability within a series of closely related protein variants complements a previous study that showed for pairs of homologous proteins from thermophilic and mesophilc organisms that raising the structural stability is the most common way (~77% of all cases) to obtain a higher thermostability [85].

Intense discussions are ongoing regarding the question if elevated protein thermostability is related to increased or decreased structural rigidity of the folded state [10, 18–23]. Part of this discussion is related to how information on structural rigidity is derived from information on mobility, in particular with respect to the temporal resolution of the experimental techniques and computational analysis [26–32]. In this context, the finding we describe here is highly relevant. As the rigidity theory-based CNA approach applied characterizes rigidity and flexibility of proteins directly, i.e., without the requirement of information on atomic movements, it does not suffer from such time dependence. Another part of the discussion is related to the fact that changes in the enthalpy, entropy and/or heat capacity can lead to thermodynamic stabilization; these changes can be linked to distinct effects on the structural stability of the folded state [19]. It was thus instructive to observe that the general increase in rigidity in the mutants of Rao *et al*. is accompanied by certain inter-helical contacts becoming weaker than in the wild type; these weakened contacts between the “modular” helices may increase the entropy of the folded state and so may further contribute to the overall stability of the systems [17, 86, 87]. This finding again calls attention to analyzing the origin of thermostability with methods that cover a wide range of temporal and spatial resolution because otherwise one effect may be hidden beneath another.

Our results are backed up with a careful validation of the accuracy and robustness of the CNA approach on the data set both from a macroscopic and microscopic point of view. As to the former, good and statistically significant correlations between experimental melting temperatures (*T*_m_) of mutants of Rao *et al*. and predicted thermodynamic thermostabilities have been found based on two independent measures (*H*_type2_ and ${\tilde{rc}}_{ij,neighbor}$), as was correctly predicted that the thermodynamic thermostability of the mutants of Reetz *et al*. is lower than that of the wild type. Furthermore, ${\tilde{rc}}_{ij,neighbor}$-based predictions of the thermodynamic thermostability on six crystal structures of wild type *Bs*LipA revealed a standard error of the mean likely within experimental error, confirming previous results of robust rigidity analyses when applying the ENT^FNC^ approach [56]. As to the latter, the detailed analysis of the unfolding pathway of wild type *Bs*LipA revealed a good agreement with respect to the early segregation of α-helices with experimental observations on other proteins with an α/β hydrolase fold. These findings are in line with previous successful applications of CNA in predicting melting temperatures and identifying structural weak spots [11–13].

From a methodological point of view, some additional comments are in order. First, in the present study we successfully predicted the thermodynamic thermostability for mutants that differ by as few as three to twelve mutations from the wild type. Compared to previous applications of CNA on either pairs of mesophilic and thermophilic homologues [12, 13] or a series of homologous proteins from different organisms living at varying temperatures [11], this finding considerably broadens the application domain of CNA towards data-driven protein engineering: There, related series of mutants with only a small number of respective mutations will be the major focus of investigations. Second, we introduced a measure for the similarity/dissimilarity of unfolding pathways of mutants and used it for explaining false thermostability predictions. We suggest to use the measure in future studies as a significance criterion to judge the reliability of thermostability predictions from CNA. Third, we introduced the median stability of rigid contacts as a new local measure for predicting thermodynamic thermostability and showed that this measure is less sensitive to details of the unfolding pathway. The measure is thus recommended for comparing thermostabilities of mutants the underlying unfolding pathways of which are dissimilar.

Finally, regarding the subset of mutants of Reetz *et al*., we find a decreased local rigidity compared to wild type, in line with findings of lower unfolding initiation temperatures, yet the mutants are more “thermostable” than the wild type in that they preserve enzymatic activity better after subjecting them to higher temperatures [42]. It would have been tempting to investigate how this relates to a potential kinetic stabilization of the mutants. However, we refrained from doing so due to the lack of direct experimental evidence for such a kinetic stabilization [49]. In turn, this finding draws attention to the fact that the term “protein thermostability” is often used in a non-discriminating sense, i.e., data reported in the literature does not allow to establish whether a protein is thermodynamically or kinetically stable [49]. This adds another layer of complexity to the question of the relation between protein thermostability and structural rigidity as it may be required to decouple observations on “increased *vs*. decreased structural rigidity” from the general description of “protein thermostability” in future studies.

## Supporting Information

S1 File The file contains additional information to the manuscript: Pairwise Pearson correlation coefficients *r* and corresponding *p* values between cluster distributions of *Bs*LipA variants from Rao *et al*. (Table A) and Reetz *et al*. (Table B), objective function of the clustering (Figure A), cluster configuration entropy *H*_type2_ *vs*. temperature obtained from the *average loss of rigidity percolation* of wild type *Bs*LipA (Figure B), correlation between predicted *T*_p_ derived from the global index *H*_type2_ and experimental thermostabilities (*T*_m_ values) of *Bs*LipA variants using single input structures. (Figure C), average loss of structural rigidity of mutant 6B during a thermal unfolding simulation (Figure D), probability density functions (PDFs) of all pairwise Pearson correlation coefficients between cluster distributions of *Bs*LipA variants (Figure E), differences in the stability of rigid contacts between wild type and variants of *Bs*LipA from Rao *et al*. (Figure F) and Reetz *et al*. (Figure G), differences in the stability of rigid contacts between variant X and wild type for selected residue neighbors (Figure H), and differences in the number of hydrogen bonds and hydrophobic tethers between *Bs*LipA mutants and wild type (Figure I).
(PDF)

S1 Video The video file shows the average loss of structural rigidity during thermal unfolding of wild type *Bs*LipA and the corresponding global rigidity index *H*_type2_ *vs*. temperature plot. See captions of Fig 3 in the main text and Figure B in S1 File for details.
(AVI)
