# Supplementary material for: Structural Rigidity and Protein Thermostability in Variants of Lipase A from Bacillus subtilis
Source: PLoS One. 2015 Jul 6;10(7):e0130289. doi: 10.1371/journal.pone.0130289 (PMC4493141; doi:10.1371/journal.pone.0130289)
Supplement: S1 File — (Table A) and Reetz et al. (Table B), objective function of the clustering (Figure A), cluster configuration entropy H type2 vs. temperature obtained from the average loss of rigidity percolation of wild type BsLipA (Figure B), correlation between predicted T p derived from the global index H type2 and experimental thermostabilities (T m values) of BsLipA variants using single input structures. (Figure C), average loss of structural rigidity of mutant 6B during a thermal unfolding simulation (Figure D), probability density functions (PDFs) of all pairwise Pearson correlation coefficients between cluster distributions of BsLipA variants (Figure E), differences in the stability of rigid contacts between wild type and variants of BsLipA from Rao et al. (Figure F) and Reetz et al. (Figure G), differences in the stability of rigid contacts between variant X and wild type for selected residue neighbors (Figure H), and differences in the number of hydrogen bonds and hydrophobic tethers between BsLipA mutants and wild type (Figure I). (PDF) [file pone.0130289.s001.pdf]

Supporting Information

Structural rigidity and protein thermostability in variants of lipase A from *Bacillus subtilis*

Prakash Chandra Rathi[1], Karl-Erich Jaeger[2,3], and Holger Gohlke[1]*

[1]Department of Mathematics and Natural Sciences, Heinrich-Heine-University, Düsseldorf, Germany

[2]Institute of Molecular Enzyme Technology, Heinrich-Heine-University, Düsseldorf, Germany

[3]Institute of Bio- and Geosciences IBG-1: Biotechnology, Research Centre Jülich, Jülich, Germany

*Universitätsstr. 1, 40225 Düsseldorf, Germany, Phone: (+49) 211-81-13662, Fax: (+49) 211-81-13847, Email: gohlke@uni-duesseldorf.de

Supplemental Methods

Body-and-bar networks

In *body-and-bar* networks, atoms are considered bodies with six degrees of freedom, and each bar between two bodies removes one degree of freedom. Depending on the strength of an interaction between two atoms, a constraint can be modeled as any number of bars between one and six with six bars completely freezing the motion between two atoms [1,2]. As done previously [3-6], covalent bonds were modeled with five (single bonds) and six (peptide and double bonds) bars, whereas hydrophobic tethers and hydrogen bonds (including salt bridges; together referred to as hydrogen bonds here) were modeled with two and five bars, respectively. A modified version of the potential by Mayo and coworkers [7] as described in ref. [8] was used to calculate hydrogen bond energies E_{HB}; hydrogen bonds with energies lower than a certain cutoff E_{cut} were included in the network (see "Thermal unfolding simulation" section in the main text for details). Hydrophobic constraints were considered between pairs of carbon and/or sulfur atoms according to a Gaussian probability function depending on the distances between the atoms (d_{ij}), the sum of their van der Waals (d_{vdw}) radii (C: 1.7 Å; S: 1.8 Å), and the full width at half maximum D_{cut} (eq. S1; see ref. [3] for details).

$$p(d_{ij}) = e^{-\frac{1}{2}\left(\frac{\left(d_{ij}-d_{vdW}\right)^2}{D_{cut}^2}\right)^2} \tag{S1}$$

Local and global rigidity indices

From a thermal unfolding trajectory, one can calculate both residue-level (local) and overall (global) rigidity characteristics of a protein [9]. Local indices can be used to investigate specific questions regarding the stability and activity of a protein. In the present study, the stability map rc_{ij} introduced by us in ref. [5] was used to characterize the local rigidity of *Bs*LipA and to understand the influence of mutations. A stability map is derived by identifying "rigid contacts" between two residues *i* and *j* that are represented by their C_α atoms. A rigid contact exists if the two residues belong to the same rigid cluster. During a thermal unfolding simulation, stability maps are then constructed in that, for each residue pair, E_{cut} (or, equivalently, a temperature derived from the relationship $T = f(E_{cut})$ described in

refs. [4,5]) is identified at which a rigid contact between these residues is lost. In that respect, the stability map is a two-dimensional itemization of the local rigidity index as detailed in ref. [9]. When filtered such that only rigid contacts between residues that are 5 Å apart from each other (measured as the distance between the closest atom pair from the two residues) are considered, a neighbor stability map results. This map helps focusing on short-range residue contacts that can be directly modulated by mutagenesis with the aim to stabilize them for improving the overall stability of a protein.

In addition, the (local) percolation index p_i introduced by us in ref. [9] was used to characterize thermal unfolding pathways of the *Bs*LipA structures. The percolation index monitors the percolation behavior (i.e., the loss of rigidity when diluting the constraint network) of a biomolecule on a microscopic level and so allows identifying the hierarchical break-down of the giant percolating cluster during a thermal unfolding simulation. The giant percolating cluster is the largest rigid cluster present at the highest E_{cut} value (i.e., at the lowest temperature at the beginning of a thermal unfolding simulation) with all constraints in place. More technically, p_i monitors the E_{cut} at which a bond segregates from the giant percolating cluster during a thermal unfolding simulation. For a C_α atom-based representation, the lower of the p_i values of the two backbone bonds is considered.

Global indices help identifying phase transition temperatures T_p at which a network switches from being largely rigid to largely flexible. Previously, we showed that T_p identified by a modified cluster configuration entropy H_{type2} [4,9] can be used for predicting the thermodynamic thermostability of and identifying structural weak spots in a protein [4-6]. The cluster configuration entropy has originally been introduced by Andraud *et al.* [10] as a morphological descriptor for heterogeneous materials and is adapted from Shannon's information theory. H_{type2} monitors the degree of disorder in the realization of a given network state: As long as a network is dominated by a very large rigid cluster, H_{type2} tends to be low because there are only a few configurations of a system with a large rigid cluster possible; H_{type2} increases when larger rigid clusters break down in smaller clusters. The H_{type2} *versus* T curve obtained from a thermal unfolding simulation was fitted with a double sigmoid [11] as done previously [6], and the temperature T_p was identified as the inflection point of the sigmoid with the larger difference in the asymptote values. This way, in most cases, a late transition involving the final decay of the giant percolating cluster is identified as T_p [5].

Supplemental Tables

Table A. Pairwise Pearson correlation coefficients *r* (upper triangle) and corresponding *p* values (lower triangle) between cluster distributions of unfolding pathways of wild type *Bs*LipA and mutants from Rao *et al*.

	**Wild type**	**TM**	**1-14F5**	**1-17A4**	**1-8D5**	**2D9**	**3-18G4**	**3-11G1**	**3-3A9**	**4D3**	**5-D**	**5-A**	**5-B**	**6B**
**Wild type**		0.38	0.54	-0.62	0.39	-0.40	-0.61	-0.61	-0.29	-0.05	0.47	0.40	0.30	-0.69
**TM**	0.280		0.90	-0.07	0.81	0.01	-0.24	-0.23	0.11	0.56	0.64	0.61	0.61	-0.31
**1-14F5**	0.108	< 0.001		-0.21	0.74	0.05	-0.21	-0.26	0.19	0.61	0.87	0.84	0.81	-0.43
**1-17A4**	0.055	0.839	0.565		-0.26	0.78	0.92	0.93	0.66	0.59	-0.09	-0.01	0.17	0.83
**1-8D5**	0.264	0.004	0.014	0.475		-0.04	-0.41	-0.46	0.22	0.32	0.46	0.43	0.36	-0.46
**2D9**	0.250	0.989	0.887	0.008	0.902		0.80	0.83	0.90	0.76	0.22	0.27	0.41	0.76
**3-18G4**	0.063	0.502	0.553	< 0.001	0.241	0.006		0.95	0.69	0.60	0.05	0.14	0.30	0.76
**3-11G1**	0.060	0.516	0.463	< 0.001	0.176	0.003	< 0.001		0.65	0.57	-0.03	0.05	0.22	0.89
**3-3A9**	0.425	0.752	0.600	0.037	0.549	< 0.001	0.029	0.042		0.73	0.35	0.41	0.48	0.48
**4D3**	0.894	0.094	0.061	0.070	0.366	0.010	0.067	0.088	0.017		0.68	0.72	0.83	0.38
**5-D**	0.173	0.046	0.001	0.805	0.179	0.539	0.892	0.943	0.322	0.032		0.99	0.96	-0.31
**5-A**	0.251	0.061	0.002	0.981	0.214	0.444	0.698	0.896	0.243	0.020	< 0.001		0.97	-0.26
**5-B**	0.402	0.060	0.004	0.631	0.309	0.234	0.394	0.541	0.159	0.003	< 0.001	< 0.001		-0.09
**6B**	0.026	0.388	0.209	0.003	0.178	0.010	0.010	0.001	0.159	0.282	0.375	0.468	0.802	
**Mean *r***	-0.06	0.29	0.34	0.28	0.16	0.41	0.29	0.27	0.43	0.56	0.40	0.43	0.49	0.12
**SEM[a]**	0.14	0.12	0.14	0.15	0.12	0.12	0.15	0.16	0.09	0.06	0.12	0.11	0.09	0.16

[a] Standard error of the mean.

Table B. Pairwise Pearson correlation coefficients *r* (upper triangle) and corresponding *p* values (lower triangle) between cluster distributions of unfolding pathways of wild type *Bs*LipA and mutants from Reetz *et al*.

	**Wild type**	**IX**	**X**	**XI**
**Wild type**		0.86	0.91	0.87
**IX**	0.001		0.87	0.79
**X**	< 0.001	0.001		0.97
**XI**	0.001	0.007	< 0.001	

Supplemental Figures

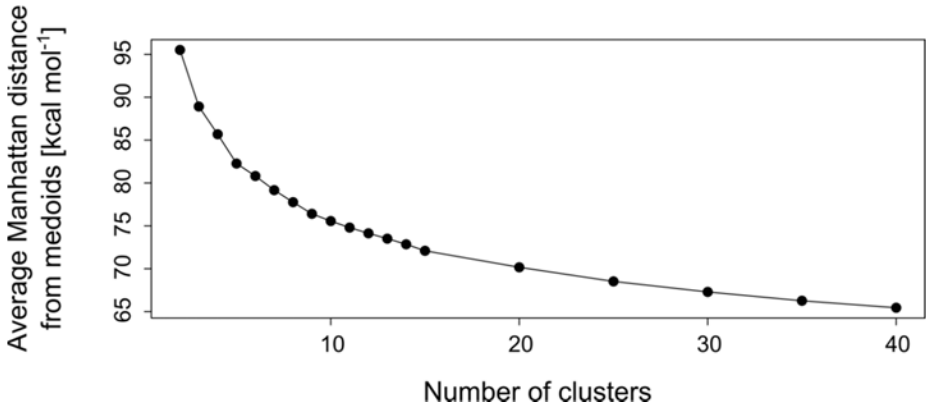

Figure A. Objective function of the clustering (the mean of the dissimilarities of all objects to their nearest medoids) *vs*. the number of clusters of p_i profiles of all *Bs*LipA variants.

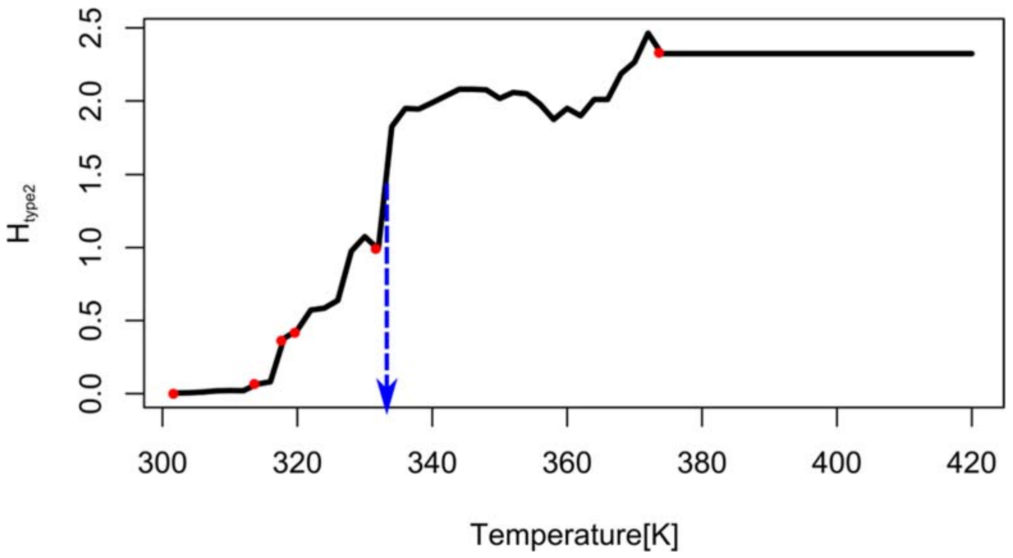

Figure B. Cluster configuration entropy H_{type2} *vs*. temperature obtained from the *average loss of rigidity percolation* over the ensemble of 2000 network topologies of wild type *Bs*LipA; this average loss of rigidity percolation is calculated from a stability map averaged over all 2000 unfolding trajectories. Steps that involve a loss of secondary structure elements during the thermal unfolding (shown in Fig. 3 in the main text) are indicated with red points. The blue arrow indicates the phase transition point on the unfolding pathway.

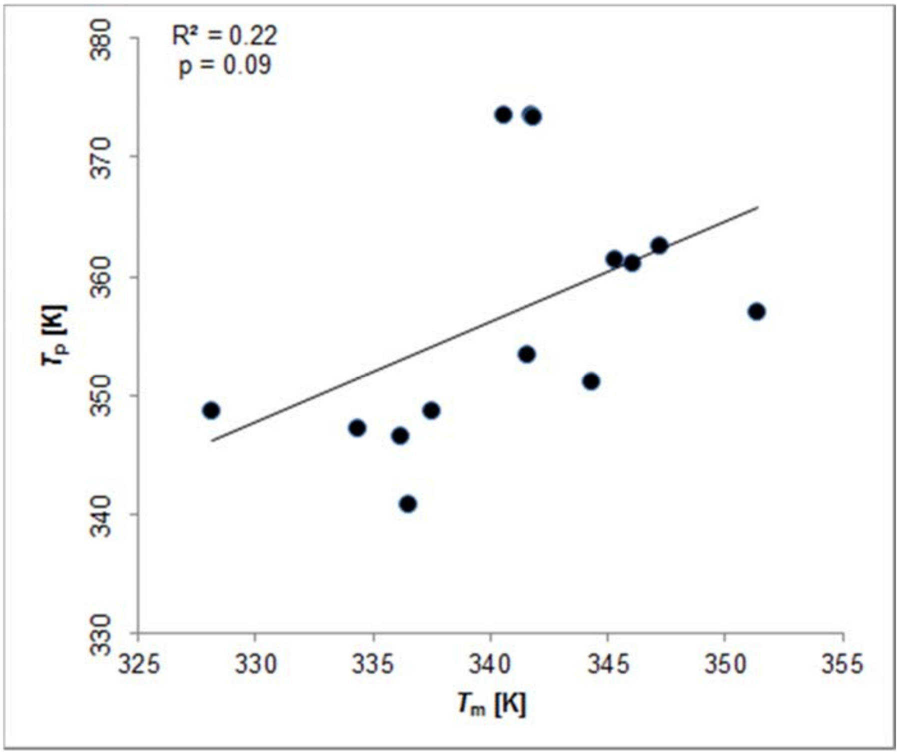

Figure C. Correlation between predicted T_p derived from the global index H_{type2} and experimental thermostabilities (T_m values) of *Bs*LipA variants using single input structures.

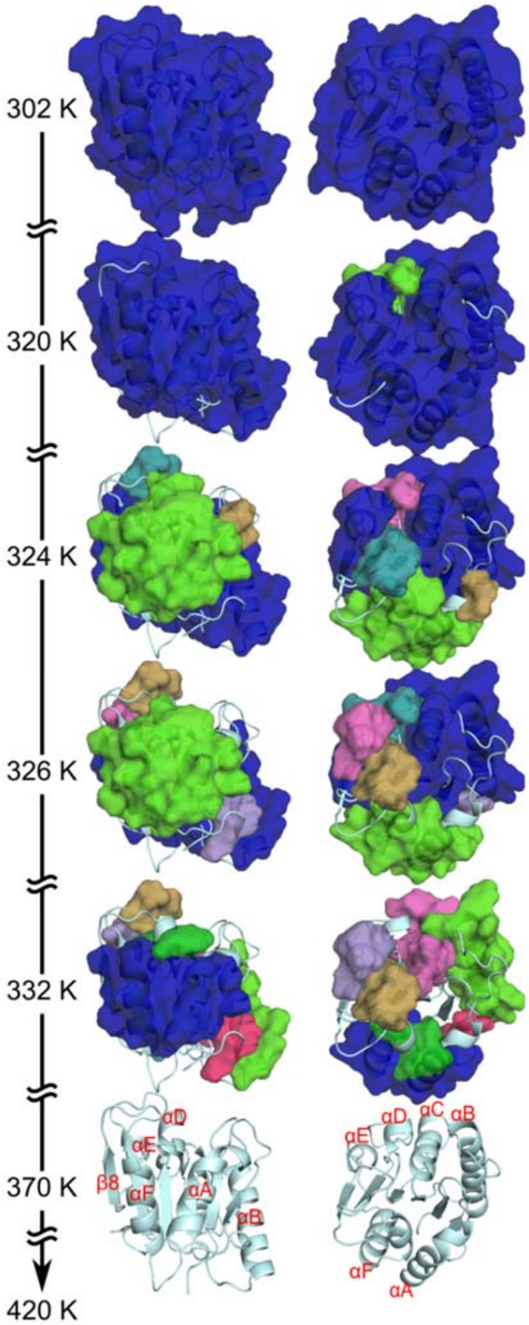

Figure D. Average loss of structural rigidity of mutant 6B during a thermal unfolding simulation. Rigid clusters are depicted as uniformly colored bodies with the largest rigid cluster always shown in blue. Temperatures are indicated for each rigid cluster decomposition depiction. At the beginning of the thermal unfolding simulation (302 K) almost the complete structure is part of the giant rigid cluster; the structure becomes completely flexible at temperatures ≥ 370 K. The right views differ from the left ones by an anti-clockwise rotation of ~90° about a horizontal axis. Important secondary structure elements are labeled.

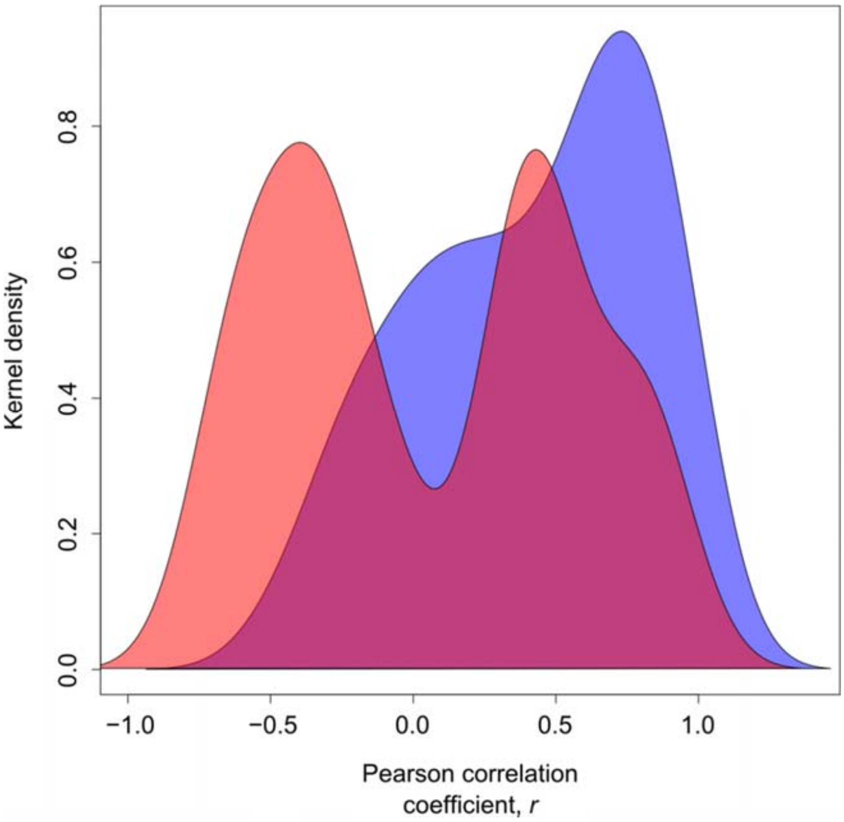

Figure E. Probability density functions (PDFs) obtained by kernel density estimation of all pairwise Pearson correlation coefficients between cluster distributions (Table) I) of all *Bs*LipA variants but the two outliers, wild type and 6B, (blue) and II) of only the two outliers (red). A normal kernel function with an optimal smoothing parameter [12] at each data point was used for calculating the PDFs.

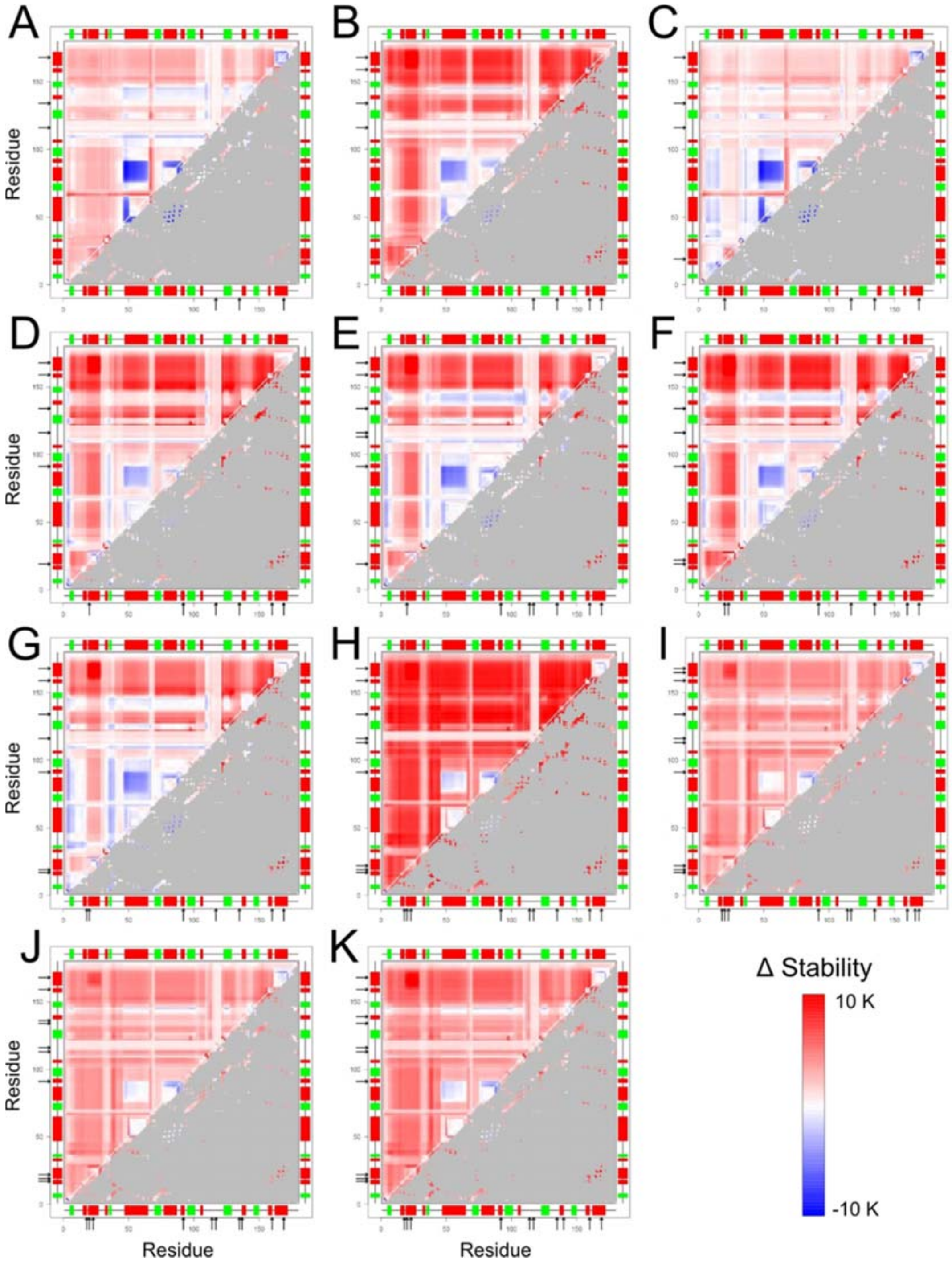

Figure F. Differences in the stability of rigid contacts between variants of *Bs*LipA from Rao *et al.* Maps depict differences (against the wild type) for mutants TM (A), 1-17A4 (B), 1-8D5 (C), 2D9 (D), 3-18G4 (E), 3-11G1 (F), 3-3A9 (G), 4D3 (H), 5-D (I), 5-A (J), and 5B (K). Secondary structure elements as computed by the DSSP program [13,14] are indicated on both abscissa and ordinate: α-helix (red rectangle), β-strands (green rectangle), loop (black line). Arrows represent the mutated residue positions.

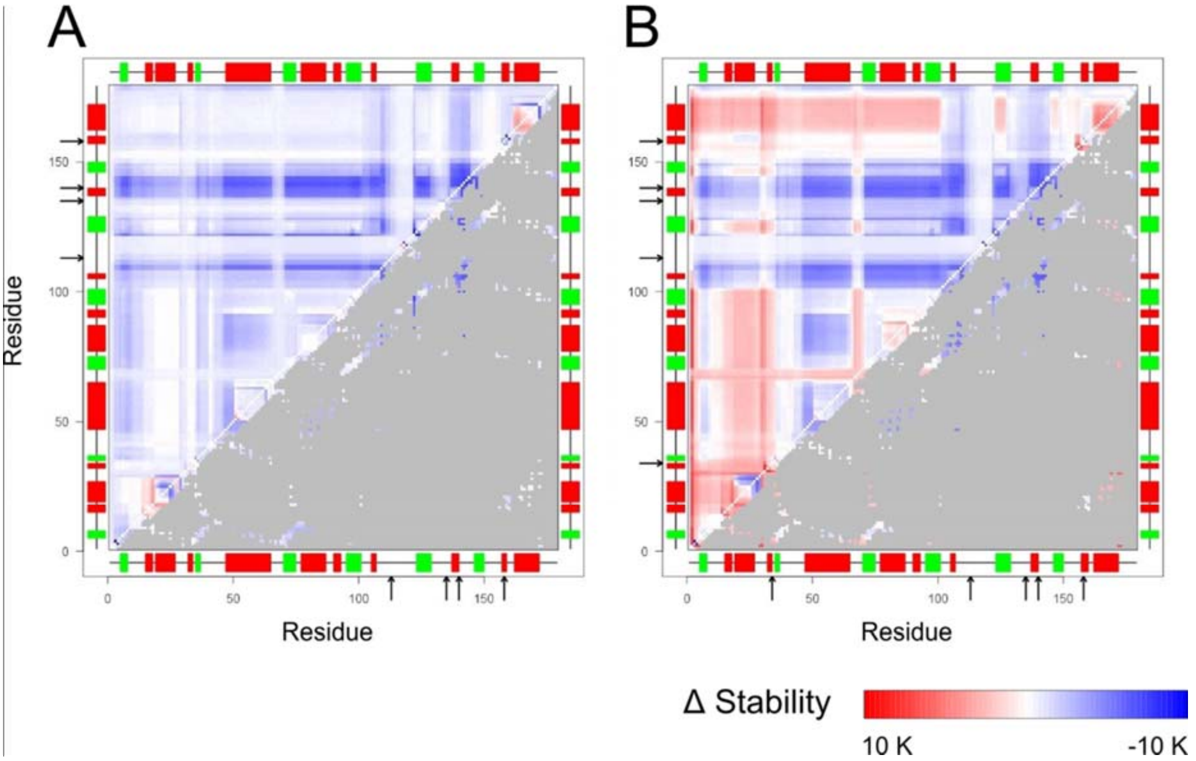

Figure G. Differences in the stability of rigid contacts between mutants of *Bs*LipA from Reetz *et al*. Maps depict differences for mutants IX (A) and XI (B) against the wild type. Secondary structure elements as computed by the DSSP program [13,14] are indicated on both abscissa and ordinate: α-helix (red rectangle), β-strands (green rectangle), loop (black line). Arrows represent the mutated residue positions of the mutants with respect to the wild type.

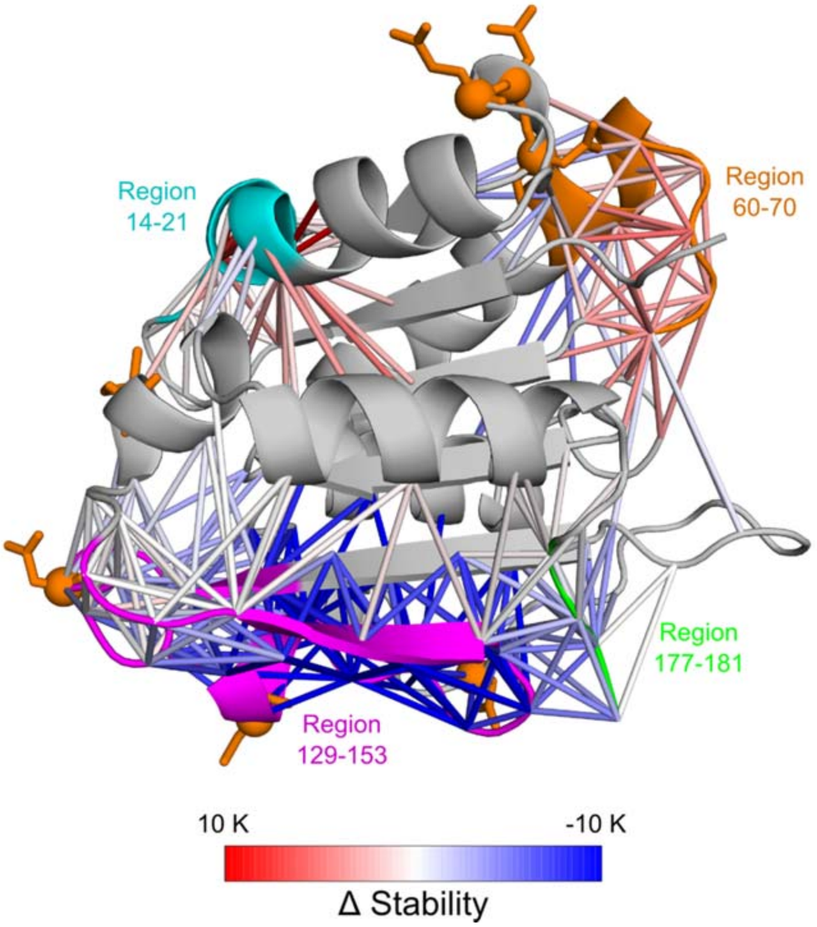

Figure H. Differences in the stability of rigid contacts between variant X and wild type for residue neighbors shown on the structure of variant X. The sticks connecting C_α atoms of residue pairs are colored according to the color scale on the bottom. A contact in red (blue) is more (less) stable in variant X than in the wild type. Only those contacts involving residues in regions discussed in the main text are shown for clarity. Mutated residues are shown as sticks and a sphere at their C_α atoms.

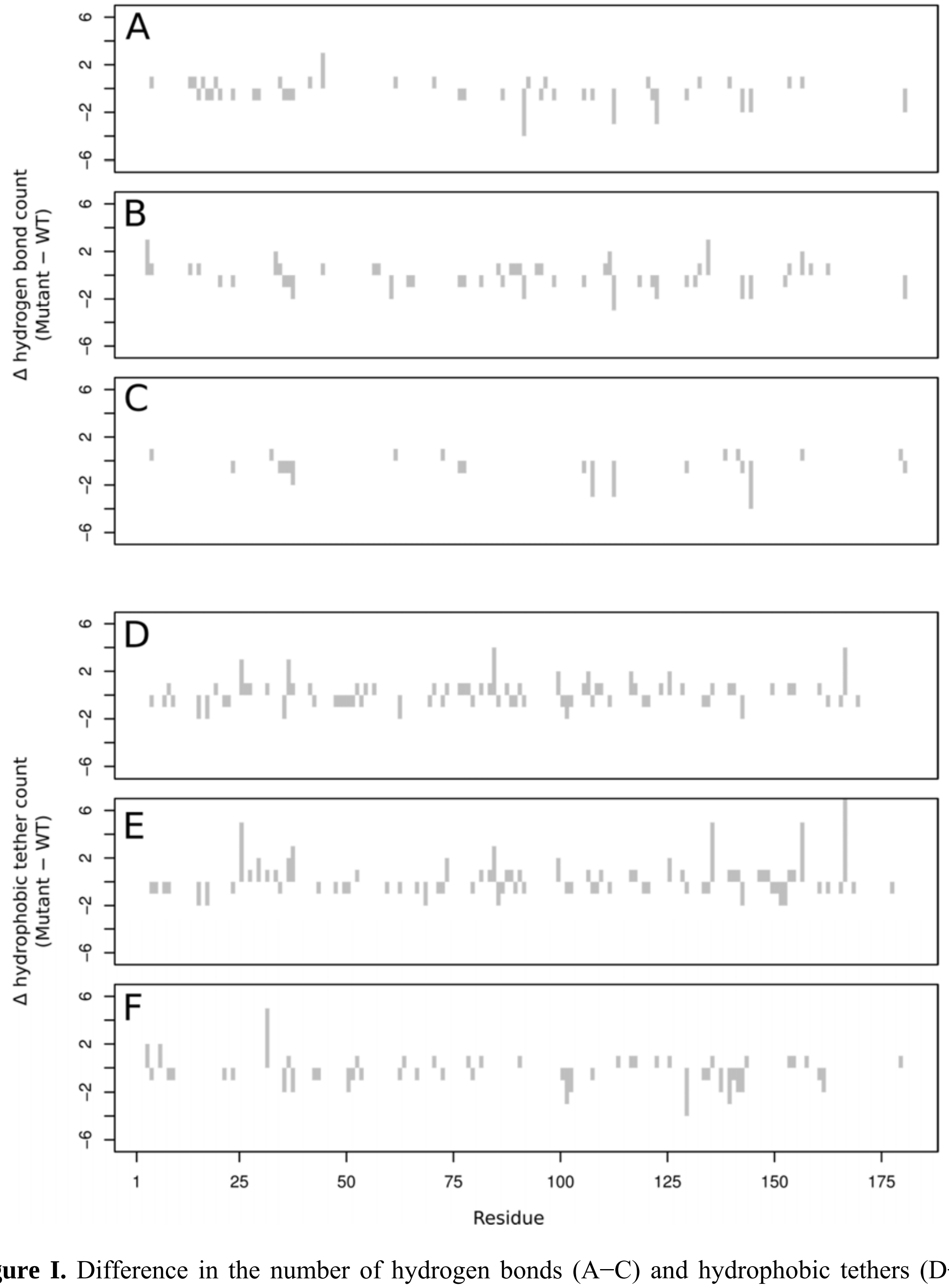

Figure I. Difference in the number of hydrogen bonds (A−C) and hydrophobic tethers (D−F) between *Bs*LipA variants (1_14F5 (A, D), 6B (B, E), and X (C, F)) *versus* WT. To focus on strong interactions, only hydrogen bonds with energies ≤ −1.0 kcal mol^{-1} were considered; similarly, for hydrophobic tethers, only hydrophobic atoms (C, S) whose van der Waals spheres are within 0.35 Å were considered.

Supplemental References

1. Whiteley W (2005) Counting out to the flexibility of molecules. Phys Biol 2: S116-S126.
2. Hespenheide BM, Jacobs DJ, Thorpe MF (2004) Structural rigidity in the capsid assembly of cowpea chlorotic mottle virus. J Phys Condens Matter 16: S5055-S5064.
3. Pfleger C, Gohke H (2013) Efficient and robust analysis of biomacromolecular flexibility using ensembles of network topologies based on fuzzy noncovalent constraints. Structure 21: 1725-1734.
4. Radestock S, Gohlke H (2008) Exploiting the link between protein rigidity and thermostability for data-driven protein engineering. Eng Life Sci 8: 507-522.
5. Radestock S, Gohlke H (2011) Protein rigidity and thermophilic adaptation. Proteins 79: 1089-1108.
6. Rathi PC, Radestock S, Gohlke H (2012) Thermostabilizing mutations preferentially occur at structural weak spots with a high mutation ratio. J Biotechnol 159: 135-144.
7. Dahiyat BI, Gordon DB, Mayo SL (1997) Automated design of the surface positions of protein helices. Protein Sci 6: 1333-1337.
8. Rader AJ, Hespenheide BM, Kuhn LA, Thorpe MF (2002) Protein unfolding: rigidity lost. Proc Natl Acad Sci U S A 99: 3540-3545.
9. Pfleger C, Radestock S, Schmidt E, Gohlke H (2013) Global and local indices for characterizing biomolecular flexibility and rigidity. J Comp Chem 34: 220-233.
10. Andraud C, Beghdadi A, Lafait J (1994) Entropic analysis of random morphologies. Physica A 207: 208-212.
11. Cairns SP, Robinson DM, Loiselle DS (2008) Double-sigmoid model for fitting fatigue profiles in mouse fast- and slow-twitch muscle. Exp Physiol 93: 851-862.
12. Silverman BW (1998) Density Estimation for Statistics and Data Analysis. London: Chapman & Hall/CRC.
13. Joosten RP, Beek TAHT, Krieger E, Hekkelman ML, Hooft RWW, et al. (2011) A series of PDB related databases for everyday needs. Nucleic Acids Res 39: D411-D419.
14. Kabsch W, Sander C (1983) Dictionary of Protein Secondary Structure - Pattern-Recognition of Hydrogen-Bonded and Geometrical Features. Biopolymers 22: 2577-2637.
